# Diabetic Retinopathy and Current Treatment Approaches: From Pathogenesis to Extracellular Vesicle-Based Therapies

**DOI:** 10.3390/ijms27146354

**Published:** 2026-07-17

**Authors:** Madina Sarsenova, Symbat Alimbek, Meruyert Imanbekova, Miras Shakhatbayev, Aidar Dairov, Murat Baidarbekov, Elvira Kadralieva, Zhanna Urazayeva, Shamrat Tlebergenov, Assel Issabekova, Vyacheslav Ogay

**Affiliations:** 1 Stem Cell Laboratory, National Center for Biotechnology, Astana 010000, Kazakhstan; sarsenova@biocenter.kz (M.S.); alimbek@biocenter.kz (S.A.); meruert.iman@gmail.com (M.I.); shakhatbayev@biocenter.kz (M.S.); dairov@biocenter.kz (A.D.); ogay@biocenter.kz (V.O.); 2 Department of General Biology and Genomics, L.N. Gumilyov Eurasian National University, Astana 010008, Kazakhstan; 3 National Scientific Center of Traumatology and Orthopedics Named After Academician N.D. Batpenov, Astana 010000, Kazakhstan; baidarbekov_m@nscto.kz; 4 Department of Ophthalmology, Non-Profit Joint-Stock Company Astana Medical University, Astana 010000, Kazakhstan; kadralieva.e@amu.kz; 5 Astromed Vision Restoration Center, Astana 010000, Kazakhstan; balmzhanna@gmail.com (Z.U.);

**Keywords:** diabetes, diabetic retinopathy, stem cell, pericyte, extracellular vesicle

## Abstract

Diabetic retinopathy (DR) is the leading cause of vision loss in early adulthood, caused by disruption of the retinal microvasculature. In the early stages of DR, there is a loss of pericytes, the cells responsible for maintaining vascular stability. Pericytes are progenitor cells and possess stem cell properties such as the potential to differentiate into various cell types, surface markers similar to mesenchymal stem cells (MSCs), and the ability to modulate the immune response. Human adipose-derived MSCs, when cultured with certain growth factors, acquire a pericyte-like phenotype, which expands the potential for pericyte applications in therapy. Current treatments for DR include anti-vascular endothelial growth factor agents, corticosteroids, and laser photocoagulation. All of these treatments are symptomatic and have adverse effects. This review covers the prevalence of DR in Kazakhstan and worldwide, the pathogenesis of the disease at the molecular and clinical level, treatment methods and modern approaches based on stem cells and pericytes.

## 1. Introduction

Diabetes is a disease of significant social importance, affecting around 463 million people worldwide. The pathogenesis of diabetes is driven by chronic hyperglycemia and the complications it causes, such as vascular damage in all organs and in the eyes. Subsequent ischemia of the retinal blood vessels triggers capillary dropout, vessel leakage and neovascularization. Diabetic retinopathy (DR) is one of the leading causes of blindness in people aged 24 to 75 with uncontrolled diabetes and remains a global health concern. It is estimated that by 2030, 191 million people worldwide will be suffering from diabetic retinopathy [1].

Depending on the presence of pathological angiogenesis, diabetic retinopathy is classified as non-proliferative DR (NPDR) or proliferative DR (PDR). NPDR is characterized by microangiogenesis, which involves the loss of pericytes and impaired endothelial cell function. Clinically, there is an increase in the permeability of the blood–retinal barrier (BRB), diabetic macular edema (DME), microaneurysms, dot–blot hemorrhages and cotton-wool spots. PDR leads to complications such as vitreous hemorrhage and traction retinal detachment [2].

The majority of treatments for retinopathy are aimed at diabetic macular edema and neovascularization. Anti-vascular endothelial growth factor (anti-VEGF) agents and corticosteroids are the main treatments for non-proliferative DR. Treatment with anti-VEGF agents requires frequent intravitreal injections, which carry a potential risk of complications. For proliferative DR, laser photocoagulation is considered the standard treatment, although it is effective in only half of cases and damages night and peripheral vision [3]. Consequently, modern treatment approaches call for a more effective and safer method of treating DR. One such method could involve the therapeutic administration of pericytes, which play a critical role in the pathogenesis of retinopathy. Pericytes are specialized mural cells that support and stabilize microvessels. Pericytes are progenitor cells and possess stem cell properties such as the potential to differentiate into various cell types, surface markers similar to mesenchymal stem cells (MSCs), and the ability to modulate the immune response, neutrophil recruitment [4], maintenance of the integrity of the blood–brain barrier [5]. MSCs have the expression of CD105, CD73, and CD90 markers and a lack of expression of the hematopoietic stem cells markers CD45, CD34, CD14, and HLA-DR according to the Tissue Stem Cell Committee [6]. Pericytes similar to MSCs differentiate to adipogenic, chondrogenic, osteogenic and myogenic lineage and present expression of CD73, CD90, and CD105 [7]. Several markers are used to verify pericyte identity, such as platelet-derived growth factor receptor-β (PDGFR-β), α-smooth muscle actin (α-SMA), neuron-glial antigen 2 (NG2), Desmin, regular of G protein signaling 5 (RGS5), and CD146 [8]. Pericytes are unable to regenerate in the mature retina; they are embedded in the basement membrane of microvessels and, together with endothelial cells, regulate vascular tone and perfusion pressure [9]. In the retina, pericytes are responsible for maintaining vascular integrity and ensuring the responsiveness of vascular cells to environmental stimuli by protecting and regulating the survival and proliferation of endothelial cells [10]. The retina is a specialized neural tissue that consists of multi-layer structures and externally lined by a single outer layer of pigmented epithelial cells [11]. During DR, the endothelial cells-to-pericytes ratio shifts from approximately 1:1 in healthy retinas to 4:1 [12]. Chronic hyperglycemia causes oxidative stress that activates nuclear factor-kB (NF-kB) and increases production of advanced glycation end products (AGEs); both mechanisms lead to pericyte loss [13]. Development of treating approaches that specifically target pericytes such as pericytes transplantation, pharmacological regulation and gene therapy on pericyte-specific pathways has been advanced in many preclinical and clinical studies [14].

The loss of pericytes has consequences that extend well beyond the vessel wall itself. As pericyte coverage declines and capillaries progressively close, regions of the retina become ischemic. This shift from adequate perfusion to hypoxia is a turning point in the disease, because it engages a transcriptional program that the retina normally reserves for low-oxygen conditions. The central mediators of this program are the hypoxia-inducible factors, principally hypoxia-inducible factor(HIF)-1α and HIF-2α. Under normal conditions, these subunits are hydroxylated and rapidly targeted for degradation, so their levels remain low. When oxygen tension falls, this degradation is halted. HIF-α accumulates, translocates to the nucleus, and partners with HIF-1β to activate a broad set of hypoxia-response genes. It is worth emphasizing that hypoxia, rather than hyperglycemia acting directly, is the more proximate trigger for this response, which is why the microvascular changes that reduce perfusion carry so much weight in the pathogenesis of the disease [15]. Among the genes induced by HIF, VEGF has attracted the most attention, and understandably so, given its central role in vascular permeability and neovascularization. However, VEGF is only one output of a much wider program, and treating it as the primary driver of disease can be misleading. A more accurate reading places VEGF downstream of HIF activation: the sequence runs from capillary loss and ischemia, to stabilization of HIF, and only then to the upregulation of VEGF and other proangiogenic and inflammatory mediators that ultimately give rise to pathological new vessels. The two principal isoforms are not interchangeable within this scheme. During retinal ischemia, they show distinct patterns of expression, with HIF-1α predominating in the neurons of the inner retina and HIF-2α concentrated in Müller glia and astrocytes, suggesting that different cell populations of the neurovascular unit mount related but not identical responses to the same hypoxic stimulus [16]. In patients with PDR, intravitreal concentrations of both HIF-1α and VEGF are elevated and rise in parallel, linking the transcription factor directly to disease severity in humans. Taken together, these observations argue that an account of DR centered on VEGF alone is incomplete. It overlooks the position of HIF as the upstream regulator that integrates the ischemic signal and coordinates the downstream angiogenic and inflammatory response and, in doing so, it overlooks a regulatory node that has itself been proposed as a therapeutic target, potentially offering broader control of the pathway than VEGF neutralization on its own [17].

Many studies have revealed that stem cells exert repairing effects mainly through the paracrine manner. Living cells secrete small extracellular vesicles (EVs) with a diameter of 30–150 nm for cell-to-cell communication and are able to regulate recipient cell activity [18]. increased studies suggest that EV treatment may be a safe and effective approach for DR therapy [19]. In this review, we have investigated the prevalence, pathogenesis and current treatment methods for DR. Particular emphasis has been placed on cell therapy using stem cells and pericytes, and cell-free therapy based on EVs for the treatment of DR.

## 2. Epidemiology of Diabetic Retinopathy

DR continues to be a common complication of diabetes mellitus (DM), affecting approximately 30–40% of people with DM, and remains a leading cause of vision impairment and blindness among working-age adults [20,21,22,23]. Of the estimated 285 million people with DM worldwide, approximately one-third have features of DR, and of these, another third have vision-threatening DR (VTDR), including clinically significant macular edema (CSME) [21]. Reported prevalence rates differ substantially across regions, populations, and diagnostic criteria, ranging from as low as 10% in some developed countries to more than 40% in certain underserved communities [23]. Among individuals with DM, the global prevalence of DR was 22.27%, 6.17% for vision-threatening DR (VTDR), and 4.07% for CSME. In 2020, the number of adults worldwide with DR, VTDR, and CSME was estimated at 103.12 million, 28.54 million, and 18.83 million, respectively. By 2045, these figures are projected to increase to 160.50 million, 44.82 million, and 28.61 million, respectively [20]. Furthermore, the highest prevalence of DR has been reported in Africa (35.90%), Southeast Asia (up to 40%), and in North America and the Caribbean (33.30%), whereas the lowest has been observed in South and Central America (13.37%) [11,14]. Additionally, Hispanic and Middle Eastern individuals with DM are more likely to have DR compared with Asian people [20]. Data on the prevalence of DR in Kazakhstan are very limited. Available evidence indicates a 27% increase in prevalence among adult patients with DM between 2015 and 2018, with higher prevalence and incidence observed in women compared to men [24]. By comparison, in the United States, 9.60 million people (26.43% of individuals with DM) were diagnosed with DR, and 1.84 million people (5.06% of individuals with DM) had VTDR in 2021 [22]. Several studies conducted in Ethiopia have shown that the prevalence of DR among patients with type 2 DM (T2DM) ranges widely from 24.35% to 36.3% [25,26]. The prevalence of DR increases with the duration of DM. In a study conducted in India, DR prevalence ranged from 5% in patients with less than 5 years of DM to 62% in those with more than 10 years [18]. According to different estimates, the prevalence of DR in India ranges from 12.5% to 26.11% [27,28,29]. In Europe, the prevalence of DR is estimated at 25.7%, while CSME is 3.7%. Furthermore, the prevalence of DR is significantly higher in people with type 1 diabetes (T1DM) (54.4%) than in those with T2DM (25.0%) [30]. However, some studies report higher prevalence rates of DR in certain European countries, for example 27.6% in Italy [31] and 28.3–36.6% in the United Kingdom [32,33], while in Germany, a comparatively low prevalence of 13.0% has been observed [34]. A comparatively low prevalence of DR has also been reported in China, ranging from 16.3% to 16.8 [35,36]. Most blindness caused by DR can be prevented by early detection and treatment of VTDR according to World Health Organization (WHO) recommendations [37].

Comparative data on the prevalence of psoriasis among patients with diabetes in a number of countries are presented in Table 1.

## 3. Diabetic Retinopathy Pathogenesis

DR begins when chronic hyperglycemia, characterized by incessant accumulation and abnormally high levels of glucose in the plasma, triggers a molecular cascade of retinal injury [38]. DR initially develops as a silent condition, with patients experiencing no visual symptoms. Over time, progressive vascular damage can lead to retinal edema and hemorrhages, ultimately resulting in impaired vision [39]. The clinical evolution of DR is currently divided into four overlapping stages: sub-clinical retinal damage, mild and moderate NPDR, severe NPDR, and proliferative DR (Table 2).

Sub-clinical retinal damage marks the earliest phase of DR. This occurs before any fundoscopic signs are visible. Hyperglycemia activates several metabolic pathways such as polyol, de novo hexosamine, protein kinase C and formation of AGEs that together overload the cell with reactive oxygen species and create a state of oxidative stress in retinal cells [40]. The oxidative burden further instigates the downstream events that worsens the early retinal neurodegeneration. In the diabetic eye, two biochemical problems are especially important in triggering the death of retinal neurons. When blood sugar is chronically high, the retina’s ability to clear glutamate, the main excitatory neurotransmitter, falls. Excess extracellular glutamate overstimulates NMDA (N-methyl-D-aspartate) receptors on neurons, leading to pathological calcium influx and subsequent excitotoxic neuronal death [41,42]. In normal condition, the healthy retina constantly produces protective proteins such as pigment-epithelium-derived factor (PEDF), somatostatin (SMS), neurotrophins (NTFs), and inter-photoreceptor retinoid-binding proteins [41]. In DR, these protective agents are downregulated, weakening the retina’s ability to counteract a toxic metabolic environment. Notably, reduced synthesis of PEDF and somatostatin, together with extracellular glutamate accumulation, is mechanistically linked to the overexpression of VEGF, a potent neuroprotective and pro-angiogenic factor, by stressed neurons [43,44]. Loss of PEDF and SMS diminishes anti-angiogenic restraint, permitting VEGF levels to rise via the HIF-1 signaling pathway, while glutamate accumulation directly stimulates VEGF gene expression [45]. This combination creates a favorable environment for VEGF upregulation, a critical event that links retinal neurodegeneration to early microvascular complications, including blood–retinal barrier breakdown and vascular leakage [46]. Paradoxically, the same metabolic disturbances that drive neuronal loss also initiate pathological angiogenic signaling, ultimately contributing to vision impairment.

**Figure 1:**
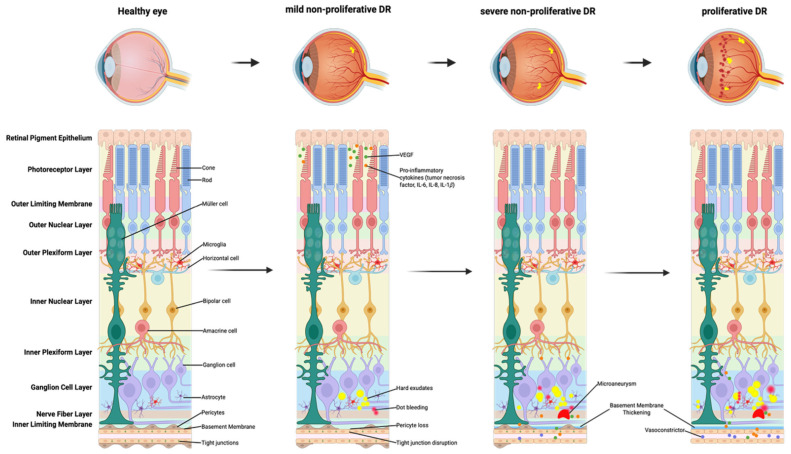
Pathogenesis and Clinical Progression of Diabetic Retinopathy. Stage 1: Subclinical Retinal Damage is marked by hyperglycemia-induced metabolic stress leading to retinal neurodegeneration and early neurovascular unit impairment, often before visible microvascular signs appear. This progresses to Stage 2: Non-Proliferative DR, where microvascular damage, including pericyte loss and basement membrane thickening, causes blood–retinal barrier breakdown, vascular leakage, and inflammation, clinically presenting as microaneurysms, hemorrhages, hard exudates, and potential macular edema. Subsequently, Stage 3: Severe Non-Proliferative DR is characterized by widespread endothelial cell loss and capillary occlusion, resulting in severe retinal ischemia, evident as “cotton wool” spots and intraretinal microvascular abnormalities. Finally, Stage 4: Proliferative DR involves profound hypoxia stimulating excessive VEGF production, inducing fragile new blood vessel formation (neovascularization), which can lead to vitreous hemorrhage and tractional retinal detachment.

In mild and moderate NPDR, the disease has moved from a sub-clinical neurodegenerative phase into a stage where structural microvascular change is now visible on examination. The earliest detectable pathological abnormality is microaneurysm formation, which defines mild NPDR. With disease progression, intraretinal dot and blot hemorrhages or bleeding emerge, marking the transition to moderate NPDR. These vascular abnormalities arise from structural and cellular damage within the retinal microvasculature, including basement membrane thickening, endothelial injury with disruption of tight junctions, and pericyte loss [47,48]. Pericytes are contractile cells that envelop capillaries and play a critical role in regulating vascular tone, maintaining capillary stability, and supporting endothelial cell survival. In DR, chronic hyperglycemia and overaction of polyol and PKC pathways induce pericyte apoptosis, leading to two major consequences: impaired local regulation of vessel caliber and blood flow, and unrestrained endothelial cell proliferation [49,50]. Together, these changes promote microaneurysm formation. Concurrently, thickening of the basement membrane and breakdown of endothelial tight junctions increase vascular permeability [51]. BRB breakdown permits the extravasation of plasma proteins, lipids, and pro-inflammatory cytokines, including interleukin-6 (IL-6), interleukin -8 (IL-8), tumor necrosis factor- α (TNF-α), and monocyte chemoattractant protein 1 [52,53] (Figure 1). These mediators, produced in part by stressed Müller cells and microglia, drive early microvascular dysfunction. Their increased levels correlate with DR severity. The continued inflammation promotes leukocyte activation and endothelial adhesion (leukostasis), resulting in progressive barrier alteration and enhanced vascular permeability [54].

As vascular leakage progresses, fluid and lipid-rich plasma constituents deposit within the retina, manifesting as hard exudates. This leads to the development of DME. Clinically, mild DME is defined by retinal thickening or hard exudates in the posterior pole that spare the central 1000 µm subfield of the macula, whereas moderate DME involves retinal thickening or hard exudates within the central subfield but not the foveal center.

As DR progresses, the previously described microvascular abnormalities become exacerbated, with a clear predominance of vasoconstriction that favors the progressions of a hypoxic retinal environment. Endothelial cell loss becomes widespread, and capillaries are progressively reduced to tubes of thickened basement membrane that are highly thrombogenic. These vessels may become occluded by platelet–fibrin aggregates or adherent leukocytes. Together, incessant vasoconstriction and capillary occlusion act as the two predominant mechanisms driving worsening retinal ischemia, culminating in the development of pre-proliferative or severe NDPR. Clinically, pre-proliferative DR is diagnosed when, without any signs of proliferative disease, the eye shows any of the following: at least 20 intraretinal hemorrhages in each of the four retinal quadrants, clear venous beading present in two or more quadrants, or intraretinal microvascular abnormalities detectable in at least one quadrant.

**Figure 2:**
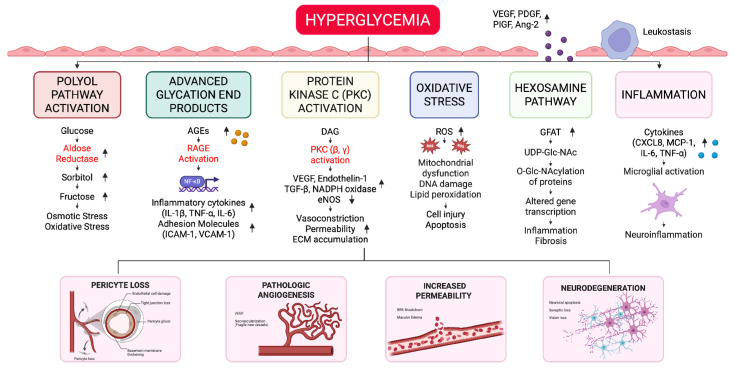
Molecular mechanisms underlying diabetic retinopathy. Chronic hyperglycemia activates multiple interconnected pathways, including the polyol, advanced glycation end-product (AGE), protein kinase C (PKC), and hexosamine pathways, leading to increased oxidative stress and inflammation. These molecular disturbances promote endothelial dysfunction, pericyte loss, and blood–retinal barrier breakdown. Enhanced production of inflammatory cytokines and angiogenic factors, particularly vascular endothelial growth factor (VEGF), further contributes to vascular leakage, leukostasis, and pathological neovascularization. In parallel, oxidative stress and neuroinflammation induce neuronal injury and apoptosis. Together, these events drive the progression of diabetic retinopathy from early microvascular damage to proliferative disease, diabetic macular edema, and ultimately, vision loss.

In PDR, long-standing metabolic injury has produced extensive capillary loss and retinal ischemia, creating a state of severe hypoxia that is the dominant driver of this stage [55]. Hypoxia shifts the balance between angiogenic and anti-angiogenic factors toward neovascular growth: hypoxic retina upregulates VEGF and other pro-angiogenic mediators while anti-angiogenic molecules such as PEDF are relatively reduced, favoring new vessel formation (Figure 2). Proteolytic enzymes released from leukocytes digest the thickened basement membrane, allowing endothelial cells to migrate out of existing vessels under the stimulus of VEGF and related factors, proliferate, and form fragile new capillaries on the retinal surface and into the vitreous cavity. These new vessels are structurally immature and weak, predisposing them to spontaneous rupture and hemorrhage; fibroblasts and extracellular matrix then grow along these new vessels, creating fibrovascular membranes that later contract and exert traction on the retina. Clinically, PDR is defined by neovascularization, either at the optic disc or elsewhere in the retina, and/or the presence of vitreous or preretinal hemorrhage. These new vessels tend to grow into the vitreous body where they are anchored by fibrovascular tissue and are highly prone to bleeding, leading to vitreous hemorrhage that can cause sudden, profound vision loss. As fibrovascular membranes contract, they can pull the neurosensory retina away from the underlying retinal pigment epithelium, producing tractional retinal detachment, a major sight-threatening complication.

Collectively, these molecular and cellular alterations lead to progressive pericyte dysfunction and loss, which represent one of the earliest and most critical events in the pathogenesis of DR. As essential components of the retinal neurovascular unit, pericytes maintain vascular stability, blood–retinal barrier integrity, and endothelial cell homeostasis through continuous bidirectional communication with endothelial cells, glial cells, and neurons. Their depletion therefore contributes not only to microvascular instability but also to neurovascular unit dysfunction, promoting inflammation, vascular leakage, and pathological angiogenesis. Consequently, therapeutic strategies aimed at preserving or restoring pericyte function have emerged as promising approaches to target the underlying disease mechanisms rather than merely treating the late manifestations of DR.

## 4. Modern Treatment Methods for Diabetic Retinopathy

**Figure 3:**
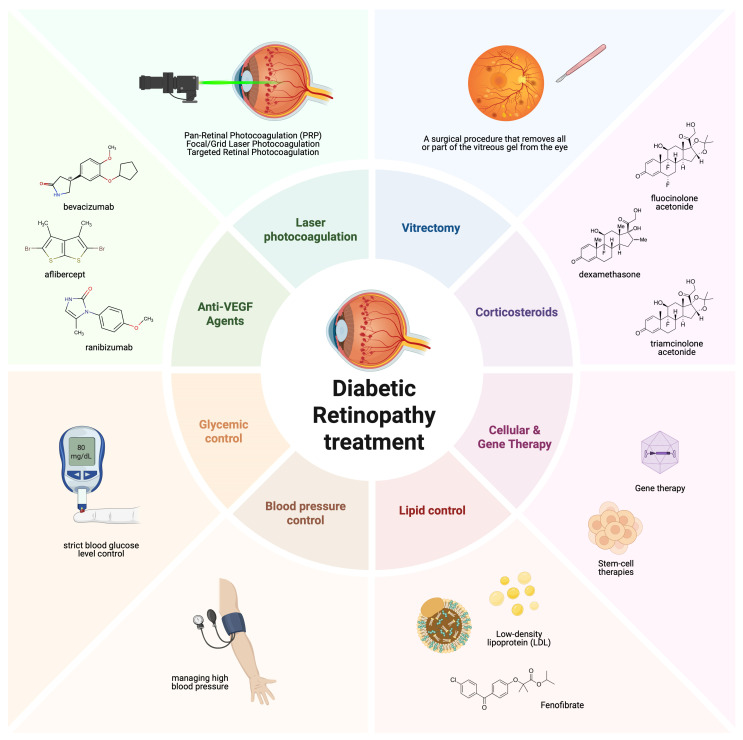
Treatment methods for diabetic retinopathy are diverse, ranging from early intervention to advanced surgical procedures, as no single approach is yet curative. Initial management focuses on rigorous systemic factor control, including tight regulation of blood glucose, blood pressure, and plasma lipids to prevent disease progression. For advanced stages, current primary treatments include intraocular injections of anti-vascular endothelial growth factor (VEGF) antibodies, such as ranibizumab and aflibercept, which are effective for proliferative DR (PDR) and diabetic macular edema (DME). Laser treatments, including pan-retinal photocoagulation (PRP) for PDR and focal/grid laser for DME, remain gold standard options, with newer subthreshold and targeted photocoagulation techniques offering improved precision. Vitrectomy is reserved for complications like long-standing vitreous hemorrhage or tractional retinal detachment. Additionally, corticosteroids like triamcinolone acetonide and dexamethasone are used for their anti-inflammatory and antiangiogenic effects, though they carry risks of cataract and glaucoma. Investigational approaches include emerging cell-based (stem-cells) and gene therapies.

Management of DR has shifted over the last two decades from purely ablative approaches toward pharmacologic and, more recently, regenerative strategies [56]. The therapeutic goals are to prevent progression from non-proliferative to proliferative disease, control DME, preserve visual function, and reduce the long-term need for invasive procedures. Previously, treatment strategies only involved the intentional destruction of the retinal tissue. Nowadays, the ultimate goal of DR treatment is to preserve visual function by preventing or delaying vision-threatening complications through stabilization of retinal neurovascular damage, while minimizing treatment load and adverse effects (Figure 3). Despite major advances with steroid implants and intravitreal drugs, important unmet needs remain, including steroid-related adverse effects, frequency of injections, incomplete responses, and limited impact on neurodegeneration and long-term microvascular remodeling [57].

Although current therapeutic approaches have substantially improved the management of DR by reducing vascular leakage and suppressing neovascularization, they primarily target downstream manifestations of the disease and do not directly restore the structural and functional integrity of the retinal neurovascular unit. Since pericyte loss precedes many of the characteristic vascular abnormalities observed in DR, increasing attention has shifted toward regenerative strategies designed to preserve pericyte viability, restore pericyte–endothelial interactions, and promote neurovascular repair.

### 4.1. Systemic and Metabolic Management

Tight control of systemic risk factors remains the foundation of DR management. Intensive regulation of blood glucose, arterial blood pressure, and serum lipid levels significantly reduces both the incidence and progression of DR. Moreover, modern antidiabetic agents such as sodium-glucose cotransporter 2 (SGLT2) inhibitors and glucagon-like peptide-1 (GLP-1) receptor agonists may provide additional microvascular protection. Nonetheless, even optimally controlled patients frequently develop sight-threatening DR with a high risk of permanent vision loss. Thus, ocular therapies are required in the majority of cases [58].

### 4.2. Intravitreal Pharmacotherapy

Anti- VEGF agents are now the mainstay for center-involving DME and for eyes with active DR at risk of progression. Anti-VEGF agents bind and neutralize VEGF, thereby reducing pathological vascular permeability and neovascularization in the diabetic retina, which in turn decreases macular edema and stabilizes or improves vision [59].

Drugs such as ranibizumab and aflibercept have demonstrated improvements in visual acuity, reductions in retinal thickness, and enhancements on the Diabetic Retinopathy Severity Scale in randomized trials [59,60,61]. However, treatment is typically administered every 4–8 weeks; thus, studies highlight substantial injection burden and a considerable proportion of partial or non-responders. In contrast, faricimab has been shown to allow a substantial proportion of patients to extend dosing intervals up to 12–16 weeks, thereby reducing the need for frequent injections. It is the first intravitreal bispecific antibody for diabetic eye disease, simultaneously inhibiting VEGF-A and angiopoietin-2 (ANG2). In the phase III YOSEMITE and RHINE trials in diabetic macular edema, faricimab achieved visual and anatomical outcomes. These studies also reported improvements in Diabetic Retinopathy Severity Scale scores, indicating a favorable effect on the underlying retinopathy [62,63,64]. Nevertheless, faricimab still requires repeated intravitreal injections. Repeated anti-VEGF treatment for diabetic macular edema still progresses to proliferative diabetic retinopathy [65]. The long-term safety and durability of the drug are less established compared to older anti-VEGF agents, and it carries the usual injection-related risks such as intraocular inflammation and raised intraocular pressure.

Intravitreal corticosteroids offer an alternative or supplementary therapy, particularly in chronic or anti-VEGF-refractory DME and in pseudophakic eyes. Dexamethasone intravitreal implants provide high-dose, short-term steroid delivery, whereas fluocinolone acetonide implants can maintain low-dose intraocular drug levels for up to three years. These implants reduce macular thickness and improve vision in selected patients but are limited by steroid-related adverse effects, primarily cataract progression and intraocular pressure elevation, requiring careful monitoring [57].

### 4.3. Emerging Pharmacologic and Device-Based Strategies

Recent pharmacologic innovation in DR has focused on extending treatment durability while maintaining efficacy, as illustrated by faricimab and high-dose aflibercept, which allow longer injection intervals yet still require repeated intravitreal administration [57,66,67]. There is still a remaining need for frequent invasive procedures. This has driven the development of sustained-release delivery devices designed to provide long-term intraocular drug exposure with fewer interventions. Sustained-release delivery devices are another important development. The Port Delivery System (PDS) with ranibizumab (Susvimo) is a refillable, surgically implanted reservoir that provides continuous intraocular anti-VEGF delivery. The PAGODA and PAVILION phase III trials in DME and DR showed that PDS refilled every 6–9 months can achieve visual and anatomic outcomes comparable to or better than frequent intravitreal injections, with markedly fewer treatment visits [68]. Although complications such as conjunctival erosion and endophthalmitis remain a concern, ongoing design modifications in surgical technique are gradually enhancing the overall safety profile [69].

In addition to VEGF, multiple molecular pathways are under investigation, including plasma kallikrein, inflammatory cytokines, and oxidative stress signaling. Fenofibrate and other peroxisome proliferator-activated receptor-α (PPARα) agonists have shown beneficial effects on DR progression in systemic trials, and nanotechnology-based drug carriers are being explored to enhance ocular bioavailability and reduce systemic exposure [70]. Overall, these approaches aim to replace high-frequency intravitreal injections with longer-acting, mechanistically diverse treatment strategies.

### 4.4. Laser Photocoagulation

In parallel with aforementioned pharmacologic and device-based advances, laser photocoagulation remains an important procedural option in the management of diabetic retinopathy, particularly in advanced stages of disease.

Laser photocoagulation is a retinal laser procedure that applies controlled thermal burns to selected areas of the retina to reduce ischemia and vascular leakage. Panretinal photocoagulation (PRP) remains a standard of care for high-risk PDR. Modern practice often integrates PRP with intravitreal anti-VEGF to reduce macular edema, facilitate regression of neovascularization, and decrease the risk of post-laser exacerbation of DME [71]. Focal or grid laser is still used for non-center-involving DME, while subthreshold micropulse laser has emerged as a tissue-sparing option that seeks to minimize scarring and visual field loss.

PRP usually provides a more durable effect compared to anti-VEGF therapy after one or a few sessions and is therefore less dependent on regular check-ups. However, laser photocoagulation is limited by permanent retinal damage with consequent peripheral visual field loss and reduced night vision, may aggravate or induce macular edema, can cause paracentral scotomas and reduced contrast sensitivity, and primarily stabilizes rather than restores vision, offering no true neurovascular regeneration.

### 4.5. Vitrectomy and Surgical Approaches

In cases where laser photocoagulation and intravitreal pharmacotherapy are insufficient or when advanced complications have already developed, surgical intervention becomes necessary. Vitrectomy and related surgical approaches are therefore reserved for more severe stages of diabetic retinopathy, particularly when vitreous hemorrhage, tractional retinal detachment or vitreomacular traction threaten or impair vision despite prior medical and laser treatment. Advances in 25- and 27-gauge systems, wide-angle viewing, and intraoperative adjuvants have improved safety and recovery profiles. Preoperative anti-VEGF is widely used to reduce intraoperative bleeding and postoperative re-proliferation [72].

In selected cases, vitrectomy may also facilitate simultaneous membrane peeling, endolaser photocoagulation, and management of coexisting macular pathology, thereby restoring or stabilizing vision where medical and laser therapies are insufficient. Nonetheless, vitrectomy and related surgery are invasive procedures with notable risks such as retinal breaks, retinal detachment, recurrent hemorrhage, cataract progression and endophthalmitis, and they require specialized surgical resources. In eyes with long-standing macular ischemia or chronic edema, visual recovery is often limited, so surgery may stabilize rather than fully restore vision [73]. Thus, there is still an unmet need for alternative treatment strategies in diabetic retinopathy.

### 4.6. Cellular and Gene-Based Approaches

Cellular and gene-based approaches are being investigated as experimental adjuncts that aim to modulate inflammation and promote neurovascular repair rather than merely suppress leakage and neovascularization. Even strategies such as stem cell-based therapies and intraocular gene delivery are at the stage of development, they reflect a shift toward more regenerative, disease-modifying treatment concepts in DR.

MSCs from bone marrow, adipose tissue, and umbilical cord have shown the ability in preclinical studies to secrete trophic and anti-inflammatory factors, stabilize the blood–retinal barrier, and protect retinal neurons from hyperglycemia-induced damage [74]. Early animal studies in DR models report reduced vascular leakage, attenuation of inflammatory markers, and improved electroretinographic parameters after intravitreal or systemic MSC administration [70].

MSC-derived EVs and exosomes are attracting attention as a cell-free alternative. These vesicles can deliver microRNAs and proteins that modulate oxidative stress, inflammation, and pathological angiogenesis in diabetic tissues, including the retina, while potentially avoiding some safety concerns associated with live cell transplantation [75]. In parallel, other cellular strategies, such as induced pluripotent stem cell-derived retinal cells, endothelial progenitor cells, and neuroprotective glial cell approaches, are under active investigation, mostly at the preclinical or early clinical stage [74].

Gene therapy for DR is less advanced than MSC therapy; however, initial studies demonstrate that intraocular vectors delivering anti-angiogenic or anti-inflammatory factors for long-term expression can be considered an adjunctive treatment method in the future [56].

In current clinical practice, DR is managed mainly through systemic risk-factor control and established ocular treatments such as intravitreal anti-VEGF agents, corticosteroid implants, laser photocoagulation, and vitreoretinal surgery (Figure 3). These interventions have substantially reduced the risk of severe vision loss but do not fully address the chronic neurovascular and inflammatory nature of the disease and still impose a considerable treatment burden. Experimental cellular and gene-based approaches, including MSC-based therapy, can therefore be used as adjuncts to modern pharmacologic approaches such as bispecific antibodies and sustained-release delivery systems for the successful treatment of DR.

### 4.7. Translational Challenges and Future Perspectives

Despite encouraging preclinical findings, the clinical translation of pericyte- and EV-based therapies remains challenging. Most available evidence is derived from in vitro experiments and rodent models, which reproduce several early features of diabetic retinopathy, including pericyte loss, vascular leakage, and inflammation, but do not fully recapitulate the complexity and chronic progression of human disease [76]. Consequently, the efficacy observed in experimental models may not directly translate to clinical outcomes.

In contrast, anti-VEGF therapy remains the current standard of care, supported by robust evidence from randomized clinical trials [62,63,64]. Therefore, future studies should evaluate whether pericyte- or EV-based therapies provide additive or disease-modifying benefits beyond existing anti-VEGF treatment rather than serving solely as alternative anti-angiogenic strategies.

Several translational challenges also need to be addressed before clinical application. These include standardized protocols for pericyte expansion and EV manufacturing, reproducible characterization of therapeutic products, optimization of ocular delivery methods, and comprehensive evaluation of long-term safety [77]. Although EVs may offer advantages over live-cell transplantation, including lower immunogenicity and easier storage, their biological activity remains highly dependent on the parental cell source, culture conditions, and isolation procedures. Addressing these limitations through standardized manufacturing and rigorous preclinical validation will be essential for successful clinical translation.

It is important to distinguish between therapeutic EVs and pathogenic EVs, as their biological effects largely depend on both the physiological state and the tissue origin of the donor cells. While EVs have demonstrated considerable therapeutic potential, EVs released under pathological conditions may actively contribute to disease progression. For example, adipose tissue-derived EVs secreted under endoplasmic reticulum stress promote endothelial dysfunction and vascular inflammation by inducing reactive oxygen species production and shifting anti-inflammatory macrophages toward a pro-inflammatory phenotype, resulting in increased secretion of IL-6, interleukin-1 β (IL-1β), and TNF-α [78]. Similarly, changes in the cellular microenvironment, such as high-glucose conditions, significantly alter exosomal cargo composition and biological activity, leading to differential effects on endothelial cell survival, oxidative stress, migration, angiogenesis, and vascular permeability [79]. These findings emphasize that the therapeutic or pathogenic properties of EVs are determined not only by their cellular origin but also by the physiological or pathological conditions under which they are produced. Furthermore, the clinical translation of therapeutic EVs is further complicated by their intrinsic heterogeneity, as EVs derived from different tissue sources may differ substantially in their protein, RNA, and lipid composition, which may influence their therapeutic efficacy [80]. Therefore, identifying the most appropriate donor cell source for a specific therapeutic application remains critical for successful clinical translation [81]. In addition to EV heterogeneity, another major challenge is the lack of standardized methods for EV isolation, purification, characterization, and quality control. Although ultracentrifugation remains the current gold standard for EV isolation, it is labor-intensive and frequently results in low purity and co-isolation of contaminants (cellular debris, proteins, or other EVs), contributing to considerable batch-to-batch variability. Therefore, standardized manufacturing and quality-control protocols are essential to ensure reproducibility and consistent therapeutic efficacy [79,81,82,83,84]. In addition, standardized dosage regimens, administration routes, measurement criteria, and efficient therapeutic cargo loading strategies have yet to be established, while the relatively low cargo content of EVs often requires repeated administration to achieve therapeutic efficacy [83,84]. Safety also remains a major concern. Although EVs are generally considered biocompatible, they can carry pathogenic cargo, including disease-associated proteins, and modified EVs may cause unexpected immunogenicity or side effects. Therefore, comprehensive preclinical studies are needed before clinical use to assess their immunogenicity, biodistribution, long-term safety, and potential toxicological effects [80,82,83]. Finally, several biological and regulatory challenges remain unresolved, including incomplete understanding of EV biogenesis, secretion and uptake mechanisms, difficulties in large-scale manufacturing, storage stability, and the absence of well-defined regulatory guidelines. Taken together, overcoming these biological, technical, and regulatory challenges will be critical to the safe and effective clinical translation of EV-based therapies [80,82,83,84].

## 5. In Vitro Studies

Given the central role of pericyte loss in DR, considerable research has focused on developing regenerative approaches capable of restoring neurovascular unit homeostasis. In particular, MSCs, pericytes, and their EVs have demonstrated the ability to modulate inflammation, preserve endothelial barrier function, attenuate oxidative stress, and promote vascular stabilization. The following section summarizes the current preclinical evidence supporting these emerging therapeutic strategies.

In this section, we focus on how these cellular and paracrine approaches affect DR pathologies, including pericyte loss, BRB breakdown, oxidative stress, and aberrant angiogenesis. The insights and new information obtained from these in vitro models lay the essential foundation for the rational design and translation of next-generation therapies for DR.

In vitro models are fundamental for describing the therapeutic mechanisms of MSCs in DR. These models typically involve culturing primary or immortalized retinal microvascular endothelial cells (HRECs/mRECs), pericytes, or retinal ganglion cells under hyperglycemic stress (high glucose, HG: 25–30 mM D-glucose for 24–72 h, with equimolar mannitol or low-glucose controls to exclude osmotic effects). MSCs from bone-marrow, adipose, or umbilical-cord sources or, more commonly, their conditioned medium (CM), purified exosomes/EVs (isolated by ultracentrifugation or precipitation and characterized per MISEV guidelines: 30–150 nm cup-shaped vesicles positive for CD9/CD63/CD81/TSG101 by Western blot, nanoparticle tracking analysis, and transmission electron microscopy) are then introduced in direct co-culture, Transwell systems, or as isolated exosomes. Multiple independent studies have consistently demonstrated that the primary mode of action is paracrine rather than direct engraftment or differentiation. MSC-derived exosomes and soluble factors mediate profound anti-inflammatory (TNF-α, IL-1β, NF-κB), anti-angiogenic (VEGF-A, ANG2, HIF-1α via Wnt/β-catenin or EGFR/Akt/mTOR suppression), barrier-protective (ZO-1, occludin, VE-cadherin; TEER, FITC-dextran permeability), antioxidant (ROS, Nrf2/HO-1), and anti-apoptotic effects, all of which are largely abolished upon exosome depletion or uptake inhibition. These findings underscore the potential of cell-free exosome-based strategies that bypass limitations of live-cell transplantation such as poor survival and immunogenicity [85].

Bone marrow-derived MSCs (BM-MSCs) remain one of the most widely studied sources for DR therapy owing to their well-documented secretome and accessibility via aspiration. In vitro evidence demonstrates that their therapeutic benefits are almost exclusively paracrine, mediated primarily by exosomes that target multiple DR hallmarks. A central mechanism is suppression of the aberrantly activated Wnt/β-catenin signaling pathway, which is upregulated under hyperglycemia and drives oxidative stress, inflammation, and pathological angiogenesis in the retinal microenvironment. BM-MSC-derived exosomes inhibit β-catenin nuclear translocation and downstream target genes, leading to decreased ROS production, lowered expression of inflammatory mediators, and reduced VEGF-driven vessel sprouting.

CM or purified exosomes from BM-MSCs protect cultured retinal endothelial cells from high-glucose (HG)-induced damage. In typical experiments, HG-stressed cells (25–30 mM D-glucose, 48–72 h) treated with BM-MSC-CM or exosomes show significant downregulation of pro-inflammatory cytokines TNF-α and IL-1β (ELISA/qRT-PCR), restoration of tight-junction proteins ZO-1 and occludin (Western blot and immunofluorescence with proper membrane localization), elevated trans-endothelial electrical resistance (TEER), and reduced paracellular permeability (FITC-dextran or albumin flux assays). These effects are largely lost when exosomes are depleted from the CM, confirming EV-mediated action. Collectively, these findings position BM-MSC exosomes as broad-spectrum modulators capable of reinforcing BRB integrity early in DR progression [70].

Adipose-derived stem cells (ASCs) provide an abundant, autologous, and minimally invasive cell source that can be harvested in large quantities from lipoaspirates. Recent in vitro advances have largely improved their therapeutic profile by directing differentiation toward a pericyte-like phenotype (P-ASCs or IADSC-PCs), thereby enhancing vascular-stabilizing paracrine signals that address the early pericyte loss characteristic of DR.

In the recent study, by Wu et al. first differentiated immortalized human ASCs (IADSCs) into pericyte-like cells using pericyte growth supplement (PGS). Successful differentiation was confirmed by robust upregulation of α-SMA and NG2 (Western blot and immunofluorescence). Exosomes were isolated from the conditioned medium of both undifferentiated IADSCs and IADSC-PCs by ultracentrifugation and fully characterized (cup-shaped morphology by TEM, 30–150 nm size by NTA, positive for CD9/CD81/TSG101 by Western blot). These exosomes were then applied to mouse retinal microvascular endothelial cells (mRECs) subjected to HG stress [79].

Fluorescence-labeled exosomes were efficiently internalized by the damaged mRECs. Compared with IADSC-Exos, IADSC-PC-Exos produced markedly superior outcomes: they reduced HG-induced apoptosis, lowered intracellular ROS levels, suppressed endothelial migration (Transwell assay) and capillary-like tube formation (Matrigel assay), and decreased monolayer permeability. At the molecular level, qRT-PCR showed strong normalization of HG-upregulated pro-angiogenic genes (VEGF-A, ANG2, MMP9) and pro-inflammatory cytokines (IL-1β, TNF-α), together with beneficial modulation of ROCK1 (cytoskeletal contractility) and CX43 (gap-junction intercellular communication). These data demonstrate that pericyte-directed preconditioning enriches the exosomal cargo for targeted vascular repair, making ASC-derived pericyte-like exosomes a promising cell-free candidate for early DR intervention [79].

Umbilical cord-derived MSCs (UC-MSCs) are particularly attractive because of their high proliferative capacity, low immunogenicity, and potent immunomodulatory secretome that can be obtained non-invasively from discarded tissue. In vitro work has highlighted their exosomes as highly effective modulators of pathological angiogenesis and inflammation in retinal endothelium.

Zhang et al. isolated exosomes from human UC-MSCs (MSC-Exos) and tested them on human retinal endothelial cells (HRECs) stimulated with VEGF-165 to recapitulate the pro-angiogenic and inflammatory milieu of DR. MSC-Exos were rapidly internalized and significantly inhibited VEGF-induced endothelial proliferation (CCK-8/EdU incorporation) and migration (scratch or Transwell assays). They also attenuated oxidative stress and markedly reduced secretion of the inflammatory cytokines TNF-α and IL-1β (ELISA) [79].

Mechanistic dissection revealed miR-218 as a key functional cargo. MSC-Exos delivered miR-218 to recipient HRECs, where they directly targeted the EGFR/Akt/mTOR signaling pathway (confirmed by Western blot of phosphorylated proteins and reporter assays). This led to coordinated downregulation of a panel of DR-associated growth factors: VEGFA, VEGFB, HIF-1α, placental growth factor (PGF), brain-derived growth factor family members, and transforming growth factor beta 1(TGF-β1). Pharmacological inhibition or miR-218 overexpression recapitulated the protective profile, while pathway reactivation reversed it, establishing EGFR/Akt/mTOR suppression as the primary axis of action. These results establish UC-MSC-Exos as a precise, miRNA-delivering platform that simultaneously curbs pathological neovascularization, oxidative damage, and inflammation in retinal vascular endothelium [86].

UC-MSCs also exert neuroprotective effects on retinal neurons, an important but often underappreciated aspect of DR pathology that frequently precedes overt vascular damage. Gao et al. isolated hUCMSC-Exos using differential ultracentrifugation and thoroughly characterized them (cup-shaped morphology by TEM, 30–150 nm diameter by NTA with peak at ~117.5 nm, positive for CD9 and CD63, negative for Calnexin by Western blot). ELISA confirmed substantial BDNF loading (2483.16 ± 281.75 pg/µg protein). Primary rat retinal neurons cultured under high-glucose conditions (35 mM D-glucose) were treated with these exosomes. hUCMSC-Exos were efficiently internalized and significantly improved neuronal survival (MTT assay) while reducing apoptosis (Annexin V/PI flow cytometry). Immunofluorescence demonstrated increased TrkB receptor expression, and all protective effects were markedly attenuated by co-treatment with a BDNF-neutralizing antibody. These results indicate that hUCMSC-Exos deliver BDNF to retinal neurons, thereby activating the BDNF-TrkB pathway and counteracting hyperglycemia-induced neuronal apoptosis. This work complements the vascular-protective actions of UC-MSC exosomes and further supports their broad therapeutic potential in DR as a neurovascular disease [87].

Further mechanistic insight into the anti-angiogenic actions of MSC-derived exosomes comes from studies on long non-coding RNA cargo. Cao et al. demonstrated that bone marrow MSC-derived exosomes transfer lncRNA SNHG7 to human retinal microvascular endothelial cells (HRMECs) under hyperglycemic stress (30 mM D-glucose, 48 h). HG exposure induced endothelial-to-mesenchymal transition, increased cell migration (Transwell), enhanced tube formation (Matrigel), and upregulated VEGF and TGF-β1. MSC exosomes carrying SNHG7 reversed these pathological changes. Mechanistically, SNHG7 acts as a molecular sponge for miR-34a-5p (which is upregulated under HG), thereby relieving miR-34a-5p-mediated repression of XBP1. Gain and loss of function experiments, RNA pull-down assays confirmed this SNHG7/miR-34a-5p/XBP1 axis. These findings reveal an additional layer of exosomal lncRNA-mediated regulation that suppresses pathological EndMT and aberrant angiogenesis, reinforcing the therapeutic value of MSC-derived exosomes in early DR [88].

Nandeesh et al. cultured primary human retinal pericytes (HRPs) under combined diabetic-like stress (high glucose 25 mM + hypoxia 1% O_2_ for 48 h). Small EVs (<200 nm) were isolated from the conditioned medium by differential ultracentrifugation and characterized by NTA, TEM, and Western blot (CD63, CD81, TSG101 positive; calnexin negative). These “stressed” EVs were then applied to healthy HRECs at a standardized particle concentration. Compared with EVs from normoxic, normal-glucose pericytes, the diabetic-condition EVs were readily internalized (PKH67 labeling) and triggered a pro-pathological phenotype in HRECs: reduced metabolic activity (MTT/CCK-8), compromised barrier function (decreased TEER, increased FITC-dextran flux), elevated migration (scratch and Transwell assays), and enhanced angiogenic sprouting (tube-formation and spheroid assays). Proteomic profiling (LC-MS/MS) of the stressed EVs identified enrichment of extracellular-matrix-remodeling proteins (e.g., MMPs, collagens) and inflammatory mediators, providing a direct mechanistic link between pericyte dysfunction and endothelial injury in early DR [89].

Lupo and colleagues generated human pericyte-like ASCs (P-ASCs) by culturing human ASCs in pericyte-specific differentiation medium containing PDGF-BB and TGF-β1 for 7–14 days. Differentiation was verified by flow-cytometry and immunofluorescence for pericyte markers (NG2, PDGFR-β, α-SMA) and loss of stemness markers. In a direct-contact or transwell co-culture system with HRECs under HG (25 mM glucose, 48–72 h), P-ASCs restored barrier function far more effectively than undifferentiated ASCs. Quantitative readouts included a statistically significant rise in trans-endothelial electrical resistance (TEER), increased protein expression and proper localization of junctional proteins VE-cadherin and ZO-1 (Western blot and immunofluorescence), and an adjuvant drop in paracellular permeability to 70-kDa FITC-dextran. Secretome analysis and ELISA further revealed that P-ASCs reduced the HG-induced secretion of TNF-α, IL-1β, and VEGF by 40–60% compared with ASC co-cultures or HG controls, demonstrating enhanced paracrine vascular stabilization when ASCs are pre-differentiated toward the pericyte lineage [90].

While most studies have investigated MSC-derived EVs, increasing evidence suggests that pericyte-derived EVs may represent a more physiologically relevant therapeutic strategy because they directly participate in retinal endothelial–pericyte communication and vascular homeostasis.

Collectively, these in vitro studies indicate that MSCs from multiple tissue origins—bone marrow, adipose tissue, and umbilical cord—confer protection against DR-relevant endothelial and pericyte dysfunction through a shared paracrine mechanism in which exosomes appear to be the dominant bioactive mediators (Table 3). BM-MSC exosomes broadly inhibit the Wnt/β-catenin pathway to coordinately suppress oxidative stress, inflammation, and pathological angiogenesis; ASCs achieve superior vascular stabilization when differentiated into pericyte-like cells, yielding exosomes that more potently normalize pro-angiogenic (VEGF-A, ANG2, MMP9) and pro-inflammatory (IL-1β, TNF-α) genes while modulating cytoskeletal (ROCK1) and gap-junction (CX43) regulators; and UC-MSC exosomes deliver specific miRNAs such as miR-218 to target the EGFR/Akt/mTOR axis and downregulate a cascade of hypoxia- and growth-factor genes (VEGFA, HIF-1α, TGF-β1). Complementary findings demonstrate that exosomes from healthy or engineered pericyte-like cells can counteract the pathogenic cargo released by stressed pericytes under diabetic conditions, restoring endothelial barrier integrity and metabolic function in vitro.

Despite this growing mechanistic catalogue, the field has not yet established which EV-associated cargos are necessary or sufficient for therapeutic efficacy, nor whether the pathways identified are broadly conserved across MSC sources and disease models. Individual studies typically isolate and validate a single candidate miR-218 [87], lncRNA SNHG7 [88], BDNF [87], or a cluster of angiogenic/inflammatory transcripts [79] using loss- or gain-of-function approaches restricted to that one axis, without excluding the contribution of the many other proteins, lipids, and RNAs co-packaged within a heterogeneous EV preparation. The apparent multiplicity of “key” mechanisms across studies may therefore reflect genuine redundancy in EV cargo, source- and model-dependent differences in cargo composition, or simply differences in which pathway each group chose to probe, rather than convergent biology. This is compounded by substantial heterogeneity in experimental design across the cited work MSC tissue origin, recipient cell type and species (human HRECs/HRMECs/HRPs versus murine mRECs), hyperglycemic stress protocols, and EV isolation/potency-assessment methods, which limits direct cross-study comparison. Head-to-head comparisons of exosomes from different MSC sources or differentiation states (e.g., pericyte-differentiated versus undifferentiated ASCs [79]) remain rare, and functional potency has generally not been benchmarked against a common reference standard. Resolving which cargos are indispensable, and whether they generalize across sources and models, will require systematic cargo depletion/enrichment or knockout-donor approaches alongside standardized, MISEV-aligned functional assays.

It is also important to note that the evidence reviewed above is derived exclusively from in vitro cell-culture systems, which do not fully recapitulate the chronic, multifactorial pathophysiology of human DR including sustained hyperglycemia over years, systemic metabolic comorbidities, immune surveillance, and the complex multicellular architecture of the human retina. Translation of these findings therefore faces several unresolved challenges. Long-term safety data are lacking: the biodistribution, immunogenicity, and theoretical tumorigenic or pro-fibrotic potential of repeated EV administration have not been characterized beyond short observation windows. Manufacturing remains a major barrier, as EV yield, cargo composition, and potency vary with donor, passage number, culture conditions, and isolation method, and no GMP-compliant, batch-consistent production and potency-release standard has been adopted across studies. Delivery is similarly unresolved; most preclinical work applies EVs directly to cultured cells, whereas clinical use would require a defined route (intravitreal injection being the most plausible for retinal targeting), dosing regimen, and evidence of adequate vitreous/retinal penetration and retention. Finally, none of the studies discussed here benchmark EV-based approaches against anti-VEGF agents (ranibizumab, aflibercept, bevacizumab), which remain the clinical standard of care for vision-threatening DR; whether the theoretical advantage of EVs simultaneously targeting inflammatory, oxidative, and barrier-protective pathways beyond VEGF alone translates into superior or complementary clinical efficacy, and at what relative cost and risk, is entirely unknown in the absence of comparative data.

## 6. In Vivo Experiments of Cell Based and Cell-Free Treatments of DR

This section of the article will examine studies investigating the therapeutic potential of stem cells and pericytes, as well as extracellular vesicles derived from these cells, using animal models of diabetic retinopathy (Table 4). There are several cell-based therapeutic strategies for treatment of DR: mesenchymal stem cells from different sources, pericytes, and their exosomes. Cell therapy using stromal cells derived from adipose tissue has shown promising results in an experimental animal model of diabetes. Recent studies have demonstrated a direct role for adipose tissue stem cells in supporting the microvasculature of the retina [91]. In a rodent model of diabetic retinopathy, adipose tissue stem cells acquired characteristics of pericytes, which suppressed proliferative angiogenesis [92]. It has been shown that the pericyte-like nature of ASCs depends on juxtacrine interactions between ASCs and endothelial cells, mediated by the NOTCH2 protein (neurogenic locus Notch homolog 2) [93].

G. Hajmousa et al. have demonstrated that adipose tissue-derived stromal cells after intravitreal injections in mice with DR function as pericytes and augment and stabilize retinal angiogenesis. CM from cultured ASC suppress high glucose-induced proinflammatory activation of bovine retinal endothelial cells in vitro [10].

Amirfarbod Yazdanyar et al. studied the hypothesis that intravitreal injection of human CD34+ stem cells harvested from bone marrow (BMSCs) can have protective effects in eyes with DR on streptozotocin (STZ)-induced diabetic mice (C57BL/6J). Subcutaneous implantation of the Alzet pump provided continuous systemic immunosuppression to avoid rejection of human cells. Human CD34+ BMSCs were harvested using magnetic beads. The eye of each mouse received an intravitreal injection of 50,000 EGFP-labeled CD34+ BMSCs or phosphate buffered saline (PBS). Simultaneous multimodal in vivo retinal imaging system consisting of fluorescent scanning laser ophthalmoscopy (angiography), optical coherence tomography (OCT) and OCT angiography was used to confirm the development of diabetic retinopathy between 5 and 6 months after induction of diabetes and study the in vivo migration of the EGFP-labeled CD34+ BMSCs in the vitreous and retina at 1 and 4 weeks following intravitreal injection. After imaging, the mice were euthanized, and the eyes were removed for immunohistochemistry. In addition, microarray analysis of the retina and retinal flat mount analysis of retinal vasculature were revealed that the expression of 162 murine retinal genes were changed. These retinal microvascular changes include areas of capillary nonperfusion and late leakage of fluorescein dye. The major molecular pathways affected by intravitreal CD34+ BMSC injection in the murine retina included pathways implicated in the pathogenesis of diabetic retinopathy including Toll-like receptor, MAP kinase, oxidative stress, cellular development, assembly and organization pathways. At 4 weeks following intravitreal injection, retinal flat mount analysis showed preservation of the retinal vasculature in eyes injected with CD34+ BMSCs when compared to PBS-injected control. The study findings support the hypothesis that intravitreal injection of human CD34+ BMSCs results in retinal homing and integration of these human cells with preservation of the retinal vasculature in murine eyes with diabetic retinopathy [94].

Mendel et al. investigated the ability of ASCs to differentiate into pericytes that can stabilize retinal vessels in oxygen-induced retinopathy and Akimba Model of Diabetic Retinopathy. ASCs express pericyte-specific markers such as α-SMA and PDGFR-β in vitro. When administered intraocularly to mice subjected to oxygen-induced retinopathy (OIR), ASCs migrated into and integrated with the retinal vasculature, maintaining expression of markers such as α-SMA and NG2 in vivo for at least 2 months. ASCs administered after OIR enhanced vascular regeneration (a 16% reduction in avascular area), while those administered before OIR prevented the reduction of retinal capillaries (a 53% reduction). Treatment of ASCs withTGF-β1 enhanced pericyte function in hASCs similar to native retinal pericytes, with increased α-SMA expression, endothelial stabilization, and microvascular protection in retinal retinal disease. Stem cell injection prevented capillary loss in diabetic Akimba mice with retinopathy (a 79% reduction 2 months after injection) [92].

Sun F. et al. investigated the mechanism by which human UC-MSC EVs alleviate DR by STZ-induced DR rat model. They found that MSC-EV injections decrease retinal apoptosis and oxidative stress. In vitro, MSC-EV administration stimulated RPE cell proliferation and increased their antioxidant capacity under HG conditions. MSC-EV improved retinal injury by delivering neuronal precursor cell-expressed developmentally downregulated 4 (NEDD4) that induced phosphatase and tensin homolog (PTEN) ubiquitination and stimulated AKT/NRF2 signaling. A new mechanism of MSC-EV-induced retinal therapeutic effects has provided a promising treatment approach for DR [95].

Gu at al. demonstrated that miR-192 participates in regulation of inflammatory response and angiogenesis by targeting the integrin subunit α1 (ITGA1) that is overexpressed in DR. ITGA1 knockdown inhibited inflammation and angiogenesis in STZ-induced diabetic retina. EVs were extracted from rat MSCs and injected into rat vitreous. Meanwhile, human retinal microvascular endothelial cells, Müller cells, and retinal pigment epithelium cells were exposed to high glucose. MSC-derived EVs relieved inflammatory response and angiogenesis by shuttling miR-192. miR-192 targeted and negatively regulated ITGA1, thereby ameliorating diabetic retinal damage. Our study established that miR-192 released by EVs from MSCs could delay the events of the inflammatory response (TNF-α, IL-6, IL-1β) and angiogenesis (VEGF, MCP-1) in DR and may represent a possible therapeutic approach for the treatment of DR [96].

Thus, numerous studies have shown that stem cells from various sources have therapeutic potential in DR and improve the clinical picture by suppressing angiogenesis, proinflammatory processes, and retinal cell apoptosis. Intravitreal administration of cells and their small extracellular vesicles in a single dose has a significant therapeutic effect within 1–2 months in an animal model of DR. Since pericytes are the primary cause of retinal abnormalities in DR, their use should enhance the therapeutic effect. Further study of the therapeutic potential of pericytes is necessary in animal models of DR.

Although numerous studies have identified candidate bioactive cargos within MSC- and pericyte-derived EVs, including microRNAs, long non-coding RNAs, proteins, and growth factors [97], the precise mechanisms responsible for their therapeutic effects remain incompletely understood. Most mechanistic studies investigate individual cargo molecules in isolated experimental systems, making it difficult to determine whether specific components or the combined action of multiple cargos mediates the observed therapeutic benefits. Furthermore, EV composition varies substantially according to the parental cell type, donor characteristics, culture conditions, and isolation methods, limiting direct comparisons between studies [98,99]. This issue is particularly relevant for pericyte-derived EVs [79,89], for which mechanistic evidence remains relatively limited. Although current data suggest that these vesicles regulate endothelial barrier integrity, inflammation, and angiogenesis, the key therapeutic cargo and signaling pathways have yet to be conclusively identified. Therefore, additional studies using standardized EV production and comprehensive multi-omics approaches are needed to establish reproducible mechanisms and facilitate clinical translation.

## 7. Clinical Trials in Diabetic Retinopathy

Despite substantial advances in the understanding and management of DR, there remains an unmet need for therapies that address the underlying mechanisms of retinal neurovascular degeneration and disease progression. Recent clinical research has therefore expanded beyond conventional pharmacological interventions to investigate regenerative and precision medicine approaches, including cell-based therapies and EV-based applications. While clinical trials of cellular therapies primarily aim to evaluate safety, feasibility, and therapeutic efficacy, clinical studies involving EVs have largely focused on their potential as minimally invasive biomarkers for disease diagnosis, staging, and prognosis. Together, these clinical investigations provide important insights into the translational potential of regenerative strategies and contribute to the evolving landscape of personalized medicine in DR. This section summarizes current clinical studies of cellular therapies and EV-based approaches, highlighting their objectives, principal findings, and implications for future clinical translation (Table 5).

### 7.1. DR Cellular Therapy Clinical Trials

Cell-based therapies have emerged as a promising frontier in ophthalmology, offering the potential to directly repair damaged retinal vasculature, preserve neuronal integrity, and modulate chronic inflammation that underpins vision loss in DR and optic [100,101,102]. Unlike conventional interventions, including anti-VEGF therapy, laser photocoagulation, and systemic glycemic optimization, which focus on symptom management or halting disease progression, cellular therapies aim to restore tissue function, leveraging autologous or allogeneic stem and progenitor cells [103,104,105]. This paradigm shift has motivated the initiation of multiple early-phase clinical trials exploring diverse cell types, delivery routes, and disease targets [106].

Intravenous administration of autologous BM- MSCs has been evaluated for both safety and preliminary efficacy in patients with non-proliferative and proliferative DR. In a pilot study of 17 patients (34 eyes), a single infusion of 3 × 10^6^ MSCs/kg demonstrated excellent tolerability over a six-month follow-up, with no infusion-related complications or serious adverse events [107]. Importantly, non-proliferative DR eyes exhibited reductions in central macular thickness and improvements in best-corrected visual acuity (BCVA), whereas proliferative DR eyes showed minimal benefit. The findings suggest that MSCs may exert paracrine-mediated anti-inflammatory and vasoprotective effects, particularly in early disease stages where residual vasculature is amenable to modulation. However, the uncontrolled nature of the study and the small sample size limit definitive conclusions regarding efficacy.

Direct ocular delivery strategies have also been investigated. To enhance local reparative potential, the Phase I trial NCT01736059 evaluated intravitreal injection of autologous CD34+ hematopoietic progenitor cells in patients with irreversible vision loss, including DR [108]. Cells were isolated under GMP conditions and delivered directly into the vitreous cavity, providing a high local concentration while minimizing systemic exposure. Six months of structured follow-up focused on ocular safety, immune response, and retinal structure. Phase I findings demonstrated the feasibility and tolerability of intravitreal autologous BM CD34^+^ cell delivery, with no major safety concerns and stable visual function over 6 months, supporting further investigation of regenerative cell therapy for ischemic and degenerative retinal disorders.

Induced pluripotent stem cells (iPSCs) have been differentiated into vascular progenitors, including endothelial cells and pericytes, in the context of DR (NCT03403699) [109]. While these cells have not yet been administered to patients, the trial focuses on establishing reproducible, clinically compliant derivation and differentiation pipelines. iPSC-derived vascular cells offer the potential for patient-specific reconstruction of the retinal microvasculature, restoring pericyte-endothelial interactions, stabilizing capillary networks, and preserving the blood–retinal barrier. By enabling autologous regenerative therapy, this approach also mitigates immunologic risks associated with allogeneic cell transplantation.

A distinct systemic approach involves allogeneic islet-cell transplantation (NCT00853424), intended to prevent DR progression by restoring endogenous insulin production and achieving physiologic glycemic control [110]. While the trial was withdrawn before patient enrollment, the protocol highlights a metabolic-oriented strategy in which cellular therapy targets the upstream drivers of microvascular disease rather than the retina directly. Such interventions exemplify the diverse therapeutic philosophies underpinning cellular approaches in ophthalmology.

Expanding beyond DR, allogeneic UC- MSCs have been investigated in NAION (NCT05147701) via combined intravenous and sub-tenon administration [111]. This Phase I trial is designed to evaluate long-term safety, tolerability, and preliminary functional outcomes over 48 months. Preclinical studies and prior clinical experience suggest UC-MSCs possess low immunogenicity and minimal tumorigenic potential. However, ocular-specific safety data, including the risk of local inflammation or ectopic tissue formation, remain to be established. This trial demonstrates the potential for immunoprivileged, off-the-shelf cellular therapies in acute optic nerve injury, expanding the regenerative scope of MSCs beyond retinal vasculopathy.

Collectively, these trials illustrate a multifaceted regenerative strategy in retinal and optic nerve disease (Table 5). MSCs primarily act via paracrine mechanisms, providing anti-inflammatory, neuroprotective, and vasoreparative effects. Intravitreal progenitor cells offer localized regeneration, while iPSC-derived vascular progenitors represent a precision medicine approach enabling patient-specific microvascular reconstruction. Allogeneic UC-MSCs provide immunoprivileged off-the-shelf therapy, particularly suited for acute neuronal injury, and islet-cell transplantation exemplifies systemic metabolic intervention.

Despite encouraging early safety data, major challenges remain. Variability in cell type, source, dose, delivery, and patient selection complicates direct comparison across studies. Long-term outcomes, engraftment stability, immune responses, and efficacy require rigorous evaluation in adequately powered, controlled trials. Additionally, harmonization of outcome measures, including structural retinal imaging, electrophysiologic testing, and functional visual assessments, is essential to facilitate translational relevance and clinical decision-making.

In conclusion, cell-based therapies in DR and optic neuropathies are at a nascent but promising stage. Early-phase trials demonstrate feasibility and short-term safety, with preliminary evidence of functional and structural improvement, particularly in early stages of disease. Future research must focus on standardized protocols, robust efficacy evaluation, and long-term safety monitoring, alongside mechanistic studies elucidating the paracrine, neuroprotective, and vasoreparative roles of each cell type. Ultimately, these therapies have the potential to shift clinical paradigms from symptomatic management toward true regenerative ophthalmology, offering the possibility of restoring retinal and optic nerve integrity in patients with currently irreversible vision loss.

**Table 5:** DR cell therapy clinical trials.

Clinical Trial	Cell Type	Delivery Route	Patient Population	Study Phase	Outcomes	Ref.
Pilot clinical trial (published study)	Autologous BM- MSCs	Intravenous (single infusion, 3 × 10^6^ cells/kg)	Patients with non-proliferative and proliferative DR	completed	Short-term safety; in NPDR eyes: decrease macular thickness & increase BCVA; no benefit in PDR eyes	[107]
2. NCT01736059	Autologous bone marrow–derived stem cells	intravitreal injection	Patients with retinal degeneration incl. DR	active	Well tolerated; demonstrated feasibility and supported further clinical evaluation	[108]
3. NCT03403699	Autologous PBMCs derived iPSCs	intravitreal injection	Patients with diabetes	Active, recruiting	Results are not published	[109]
4. NCT00853424	islet cell	transplantation	Patients with DR	Withdrawn	Results are not published	[110]
5. NCT05147701	allogeneic adult UC-MSCs	intravenous and sub-tenon delivery	non-arteritic ischemic optic neuropathy including DR	active	Results are not published	[111]

### 7.2. EV Clinical Trials in Diabetic Retinopathy

To date, clinical investigation of EVs in DR remains limited to observational studies, with no completed interventional trials demonstrating therapeutic efficacy. The available NIH-registered studies primarily focus on EV cargo profiling across disease stages to identify biomarkers of retinal microvascular injury and neurovascular unit dysfunction.

One key line of investigation focuses on systemic EV profiling to identify circulating biomarkers reflective of diabetic retinopathy pathophysiology and disease progression. In this context, a plasma exosomal proteomics study evaluates individuals ranging from healthy controls to patients with diabetes without retinopathy, as well as those with non-proliferative and proliferative DR. By comparing EV protein signatures across these groups, the study seeks to identify differentially expressed proteins associated with disease onset and progression, thereby providing candidate biomarkers for early detection and staging of DR [112].

Complementing this systemic approach, another study extends EV profiling to ocular compartments by analyzing EVs derived from plasma, aqueous humor, and vitreous fluid in patients with proliferative DR undergoing vitrectomy. This multifluid design enables a more direct assessment of the intraocular microenvironment, with the aim of linking EV protein signatures to advanced pathological features such as retinal ischemia, neovascularization, and fibrotic remodeling [113].

In parallel, a third line of research focuses on EV-associated nucleic acids, particularly circulating microRNAs. Serum-derived EV miRNA profiling in patients with DR is being used to identify disease-associated regulatory RNA signatures that may reflect endothelial dysfunction, inflammatory activation, and retinal cell stress. These findings support the concept that EVs act as stable circulating carriers of molecular information that mirrors retinal pathology [114].

Collectively, these complementary approaches consistently demonstrate that EV cargo is closely associated with the underlying pathophysiology of DR, including endothelial activation, pericyte loss, and chronic inflammation. However, despite these promising biomarker signals, all currently available clinical studies remain limited to observational designs. No interventional EV-based clinical trials in DR have yet reported therapeutic outcomes, underscoring a substantial gap between mechanistic and preclinical advances and their translation into clinical therapy.

## 8. Conclusions

Treatment for diabetic retinopathy is currently symptomatic and does not address the underlying cause of the disease. Furthermore, all current treatments have side effects and require more therapeutic options. Stem cell therapy is an advanced treatment method with fewer side effects. EVs are currently being developed that possess host cell properties and carry information to recipient cells. These EVs contain proteins, RNAs, and microRNAs from the host cells. Research into the therapeutic efficacy of these vesicles is currently gaining popularity. MicroRNAs have been participated in regulation of pericyte survival in DR. MiR-195 stimulate pericytes death by binding to Sirtuin 1 (SIRT1) in diabetic eyes [115]. MiR-126 has been implicated in protection of retinal pericytes by targeting to the p38 MAPK/NF-kB signaling pathway [116]. In addition to the fact that certain microRNAs play a key role in the pathogenesis of DR, small extracellular vesicles carrying microRNAs from pericytes retain the properties of the host cells and can therefore be used in place of pericytes for the treatment of DR. The development of such safe and effective approaches may be the future for DR treatments.

## Figures and Tables

**Table 1:** Summary of prevalence rates of diabetic retinopathy among individuals with diabetes mellitus in different countries.

Country	Prevalence of DR %	References
Kazakhstan	27.00	[24]
United States	26.43	[22]
Ethiopia	24.35–36.30	[25,26]
India	12.50–26.11	[27,28,29]
Italia	27.6	[31]
United Kingdom	36.6	[32,33]
Germany	13.0	[34]
China	16.3–16.8	[35,36]

**Table 2:** Classification of diabetic retinopathy stages.

Diabetic Retinopathy Stages	Pathophysiology	Clinical Characteristics
No DR	No abnormalities observed	No abnormalities observed
Mild non-proliferative DR	Early microvascular damage limited to formation of microaneurysms	Isolated microaneurysms on fundus photography; no other lesions such as hemorrhages, hard exudates, or cotton-wool spots.
Moderate non-proliferative DR	Progressive endothelial injury with additional leakage and capillary compromise, but still without the extensive ischemia that defines severe disease.	Presence of any of the following: microaneurysms, retinal dot- and blot-hemorrhages, hard exudates or cotton-wool spots; however, the severe-NPDR criteria are not met.
Severe non-proliferative DR	Marked capillary loss and widespread retinal hypoxia; the balance shifts toward angiogenic signaling (VEGF) but neovascularization has not yet appeared.	≥20 intraretinal hemorrhages in each of the four quadrants or definite venous beading in ≥2 quadrants or intraretinal microvascular abnormalities (IRMA) in ≥1 quadrant; cotton-wool spots are common.
Proliferative DR	Chronic ischemia drives robust VEGF-mediated neovascularization; new vessels proliferate on the optic disc or elsewhere, become fragile, and may be accompanied by fibrovascular tissue that contracts.	Neovascularization of the disc or elsewhere; vitreous or preretinal hemorrhage; fibro-vascular membranes that can cause tractional retinal detachment; possible iris neovascularization (rubeosis) leading to neovascular glaucoma.

**Table 3:** Summary of key in vitro studies on MSC-, pericyte-like cell-, and exosome/EV-based approaches in diabetic retinopathy models.

Study (Authors, Year)	MSC/Pericyte Source	Model and Conditions	Key Intervention	Main Findings	Primary Mechanism(s)	Ref.
Kąpa et al. (2025)	Bone marrow MSCs (BM-MSCs)	HG-stressed retinal endothelial cells	BM-MSC exosomes or conditioned medium	↓ oxidative stress, inflammation & angiogenesis; ↑ ZO-1/occludin expression, TEER & BRB integrity	Suppression of Wnt/β-catenin signaling pathway	[70]
Wu et al. (2024)	Immortalized ASCs differentiated into pericyte-like (IADSC-PCs)	Mouse retinal microvascular ECs (mRECs) + high glucose (HG)	Exosomes from IADSC-PCs vs. undifferentiated ASCs	Superior reduction in apoptosis, ROS, migration, tube formation & permeability; normalized VEGF-A, ANG2, MMP9, IL-1β, TNF-α, ROCK1, CX43	Pericyte differentiation enriches exosomal cargo for vascular stabilization	[79]
Zhang et al. (2025)	Umbilical cord MSCs (UC-MSCs)	Human retinal ECs (HRECs) + VEGF-165	MSC-Exos	↓ proliferation, migration, oxidative stress & TNF-α/IL-1β secretion; ↓ VEGFA, HIF-1α, TGF-β1 etc.	Delivery of miR-218 targeting EGFR/Akt/mTOR pathway	[87]
Lupo et al. (2023)	Adipose MSCs differentiated into pericyte-like (P-ASCs)	Human retinal ECs (HRECs) + HG (25 mM)	Direct or Transwell co-culture with P-ASCs vs. undifferentiated ASCs	P-ASCs superior: ↑ TEER (up to 73%), ↑ VE-cadherin/ZO-1, ↓ TNF-α/IL-1β/VEGF (40–60%)	Enhanced PDGF-B/PDGFR-β paracrine signaling & BRB stabilization	[90]
Nandeesh et al. (2025)	Human retinal pericytes (stressed)	Human retinal ECs (HRECs)	Small EVs from HG + hypoxia-stressed pericytes	↓ metabolic activity & barrier function; ↑ permeability, migration & angiogenesis; enriched ECM/inflammatory proteins	Pathological pericyte-to-endothelium EV transfer drives DR progression (contrasts therapeutic EVs)	[89]

**Table 4:** Summary of key in vitro studies on cell-base/EV-based approaches in diabetic retinopathy models.

No.	Cell Types	Amount, Injection Type	Signaling Pathway	Effect	Diabetic Model, Number of Animal, Mothods	Ref.
1	Adipose tissue-derived stromal cells	10,000 ASCs in 1 µL	In ROP retinas of ASC-treated mice, expression of Angpt1 and Fgf2 were increased, while level of Angpt2 and Vegfa were reduced	ASCs activate retinal angiogenesis in ROP mice via acquisition of a pericytes position and function	Hypoxia-driven angiogenesis, male C57BL/6J mice	[10]
2	CD34+ bone marrow stem cells (hematopoietic stem cells and endothelial progenitor cells)	intravitreally with 50,000 EGFP-labeled CD34+ MSCs	Toll-like receptor, MAP kinase, oxidative stress, cellular development, assembly and organization pathways	At 4 weeks following intravitreal injection, retinal flat mount analysis showed preservation of retinal vasculature	Streptozotocin-induced (C57BL/6L) mice, DR between 5 and 6 months after induction of diabetes	[94]
3	hASC-derived pericytes	after OIR intravitreally injection of 10,000 hASCs at P12; 1000 mASCs at P9 or 10,000 hASCs into 5 weeks old Akimba	Treatment with TGF-β1 enhanced hASC pericyte function	hASC-derived pericytes integrate with retinal vasculature and enhance vessel regrowth in OIR, prevent capillary loss in Akimba mouse.	OIR on NOD SCID mice, Akimba mouse model of diabetic retinopathy	[92]
4	(UC-MSC-EVs	Intravitreal injection of 10^6^ particles in 3 µL	MSC-sEV-delivered NEDD4 promoted PTEN ubiquitination and activated AKT/NRF2 signaling	NEDD4 could prevent DR progress	STZ (35 mg/kg) established diabetic rat model	[95]
5	Rat adipose mesenchymal stem cells-derived extracellular vesicles	Intravitreal injection of 5, 10, 20 and 40 µg/mL Evs	miR-192 in the Evs play a main role in inducing inflammation as well as angiogenesis by targeting ITGA1	MSC-derived Evs inhibit inflammation and angiogenesis in vitro and in vivo.	STZ-induced (65 mg/kg) diabetic rat model	[96]

## Data Availability

No new data were created or analyzed in this study. Data sharing is not applicable to this article.

## Abbreviations

The following abbreviations are used in this manuscript:

AGEs	Advanced-glycation end-products
DR	Diabetic retinopathy
DM	Diabetes mellitus
DME	Diabetic macular edema
VTDR	Vision-threatening diabetic retinopathy
CSME	Clinically significant macular edema
NPDR	Non-proliferative DR
PDR	Proliferative diabetic retinopathy
T2DM	Type 2 diabetes mellitus
T1DM	Type 1 diabetes mellitus
VEGF	Vascular endothelial growth factor

## References

[1] Quiroz J., Yazdanyar A. (2021). Animal model of diabetic retinopathy. Ann. Transl. Med..

[2] Cheung N., Mitchell P., Wong T.Y. (2010). Diabetic retinopathy. Lancet.

[3] Heng L.Z., Comyn O., Peto T., Tadros C., Ng E., Sivaprasad S., Hykin P.G. (2013). Diabetic retinopathy: Pathogenesis, clinical grading, management and future developments. Diabet. Med..

[4] Ayres-Sander C.E., Lauridsen H., Maier C.L., Sava P., Pober J.S., Gonzalez A.L. (2013). Transendothelial Migration Enables Subsequent Transmigration of Neutrophils through Underlying Pericytes. PLoS ONE.

[5] Nakagawa S., Deli M.A., Kawaguchi H., Shimizudani T., Shimono T., Kittel Á., Tanaka K., Niwa M. (2009). A new blood–brain barrier model using primary rat brain endothelial cells, pericytes and astrocytes. Neurochem. Int..

[6] Dominici M., Le Blanc K., Mueller I., Slaper-Cortenbach I., Marini F., Krause D., Deans R., Keating A., Prockop D., Horwitz E. (2006). Minimal criteria for defining multipotent mesenchymal stromal cells. The International Society for Cellular Therapy position statement. Cytotherapy.

[7] Ahmed T.A., Shousha W.G., Abdo S.M., Mohamed I.K., El-Badri N. (2020). Human Adipose-Derived Pericytes: Biological Characterization and Reprogramming into Induced Pluripotent Stem Cells. Cell. Physiol. Biochem..

[8] Hazrati R., Davaran S., Keyhanvar P., Soltani S., Alizadeh E. (2024). A Systematic Review of Stem Cell Differentiation into Keratinocytes for Regenerative Applications. Stem Cell Rev. Rep..

[9] Caporarella N., D’Angeli F., Cambria M.T. (2019). Pericytes in microvessels: From “mural” function to brain and retina regeneration. Int. J. Mol. Sci..

[10] Hajmousa G., Przybyt E., Pfister F., Paredes-Juarez G.A., Moganti K., Busch S., Kuipers J., Klaassen I., van Luyn M.J.A., Krenning G. (2018). Human adipose tissue-derived stromal cells act as functional pericytes in mice and suppress high-glucose-induced proinflammatory activation of bovine retinal endothelial cells. Diabetologia.

[11] (2021). GBD 2019 Blindness and Vision Impairment Collaborators; Vision Loss Expert Group of the Global Burden of Disease Study. Causes of blindness and vision impairment in 2020 and trends over 30 years, and prevalence of avoidable blindness in relation to VISION 2020: The Right to Sight: An analysis for the Global Burden of Disease Study. Lancet Glob. Health.

[12] Arboleda-Velasquez J.F., Valdez C.N., Marko C.K., D’Amore P.A. (2015). From pathobiology to the targeting of pericytes for the treatment of diabetic retinopathy. Curr. Diabetes Rep..

[13] Trudeau K., Molina A.J., Roy S. (2011). High glucose induces mitochondrial morphology and metabolic changes in retinal pericytes. Investig. Ophthalmol. Vis. Sci..

[14] D’Esposito F., Cappellani F., Visalli F., Capobianco M., Rapisarda L., Avitabile A., Cannizzaro L., Malaguarnera R., Gagliano G., Maniaci A. (2025). Pericytes as Key Players in Retinal Diseases: A Comprehensive Narrative Review. Biology.

[15] Kurihara T., Westenskow P.D., Friedlander M. (2014). Hypoxia-inducible factor (HIF)/vascular endothelial growth factor (VEGF) signaling in the retina. Adv. Exp. Med. Biol..

[16] Wang X., Wang G., Wang Y. (2009). Intravitreous vascular endothelial growth factor and hypoxia-inducible factor 1a in patients with proliferative diabetic retinopathy. Am. J. Ophthalmol..

[17] D’Amico A.G., Maugeri G., Reitano R., Bucolo C., Saccone S., Drago F., D’Agata V. (2015). PACAP Modulates Expression of Hypoxia-Inducible Factors in Streptozotocin-Induced Diabetic Rat Retina. J. Mol. Neurosci. MN.

[18] Zhai M., Zhu Y., Yang M., Mao C. (2020). Human Mesenchymal Stem Cell Derived Exosomes Enhance Cell-Free Bone Regeneration by Altering Their miRNAs Profiles. Adv. Sci..

[19] Mathew B., Ravindran S., Liu X., Torres L., Chennakesavalu M., Huang C.C., Feng L., Zelka R., Lopez J., Sharma M. (2019). Mesenchymal stem cell-derived extracellular vesicles and retinal ischemia-reperfusion. Biomaterials.

[20] Teo Z.L., Tham Y.-C., Yu M., Chee M.L., Rim T.H., Cheung N., Bikbov M.M., Wang Y.X., Tang Y., Lu Y. (2021). Global prevalence of diabetic retinopathy and projection of burden through 2045: Systematic review and meta-analysis. Ophthalmology.

[21] Lee R., Wong T.Y., Sabanayagam C. (2015). Epidemiology of diabetic retinopathy, diabetic macular edema and related vision loss. Eye Vis..

[22] Lundeen E.A., Burke-Conte Z., Rein D.B., Wittenborn J.S., Saaddine J., Lee A.Y., Flaxman A.D. (2023). Prevalence of diabetic retinopathy in the US in 2021. JAMA Ophthalmol..

[23] Mohammed M.R., Fathi S.A. (2025). Global prevalence of diabetic retinopathy over the last decade (2015–2025): A scoping review. SSRN.

[24] Beisembinova N., Kosherbayeva L., Balmukhanova A., Hailey D., Tolganbayeva K., Seyduanova L. (2021). Diabetes and diabetic retinopathy in Kazakhstan from 2013–2018. Curr. Pediatr. Res..

[25] Wondmeneh T.G., Mohammed J.A. (2024). Prevalence of diabetic retinopathy and its associated risk factors among adults in Ethiopia: A systematic review and meta-analysis. Sci. Rep..

[26] Zegeye A.F., Temachu Y.Z., Mekonnen C.K. (2023). Prevalence and factors associated with Diabetes retinopathy among type 2 diabetic patients at Northwest Amhara Comprehensive Specialized Hospitals, Northwest Ethiopia 2021. BMC Ophthalmol..

[27] Mehta R., Punjabi S., Bedi N. (2020). Prevalence of diabetic retinopathy: A tertiary care centre based study. Indian J. Clin. Exp. Ophthalmol..

[28] Raman R., Vasconcelos J.C., Rajalakshmi R., Prevost A.T., Ramasamy K., Mohan V., Mohan D., Rani P.K., Conroy D., Das T. (2022). Prevalence of diabetic retinopathy in India stratified by known and undiagnosed diabetes, urban-rural locations, and socioeconomic indices: Results from the SMART India population-based cross-sectional screening study. Lancet Glob. Health.

[29] Vashist P., Senjam S.S., Gupta V., Manna S., Gupta N., Shamanna B.R., Bhardwaj A., Kumar A., Gupta P. (2021). Prevalence of diabetic retinopahty in India: Results from the National Survey 2015-19. Indian J. Ophthalmol..

[30] Li J.Q., Welchowski T., Schmid M., Letow J., Wolpers C., Pascual-Camps I., Holz F.G., Finger R.P. (2020). Prevalence, incidence and future projection of diabetic eye disease in Europe: A systematic review and meta-analysis. Eur. J. Epidemiol..

[31] Vujosevic S., Midena E. (2016). Diabetic retinopathy in Italy: Epidemiology data and telemedicine screening programs. J. Diabetes Res..

[32] Mathur R., Bhaskaran K., Edwards E., Lee H., Chaturvedi N., Smeeth L., Douglas I. (2017). Population trends in the 10-year incidence and prevalence of diabetic retinopathy in the UK: A cohort study in the Clinical Practice Research Datalink 2004–2014. BMJ Open.

[33] Scanlon P.H., Nevill C.R., Stratton I.M., Maruti S.S., Massó-González E.L., Sivaprasad S., Bailey C., Ehrlich M., Chong V. (2022). Prevalence and incidence of diabetic retinopathy (DR) in the UK population of Gloucestershire. Acta Ophthalmol..

[34] Ponto K.A., Koenig J., Peto T., Lamparter J., Raum P., Wild P.S., Lackner K.J., Pfeiffer N., Mirshahi A. (2016). Prevalence of diabetic retinopathy in screening-detected diabetes mellitus: Results from the Gutenberg Health Study (GHS). Diabetologia.

[35] Hou X., Wang L., Zhu D., Guo L., Weng J., Zhang M., Zhou Z., Zou D., Ji Q., Guo X. (2023). Prevalence of diabetic retinopathy and vision-threatening diabetic retinopathy in adults with diabetes in China. Nat. Commun..

[36] Wang J., Zhang H. (2024). Prevalence of diabetic retinopathy and its risk factors in rural patients with type 2 diabetes referring to Beijing Huairou Hospital, China. BMC Ophthalmol..

[37] Bascaran C., Zondervan M., Walker C., Astbury N.J., Foster A. (2022). Diabetic retinopathy in Africa. Eye.

[38] Morya A.K., Ramesh P.V., Nishant P., Kaur K., Gurnani B., Heda A., Salodia S. (2024). Diabetic retinopathy: A review on its pathophysiology and novel treatment modalities. World J. Methodol..

[39] Mounirou B.A.M., Adam N.D., Yakoura A.K.H., Aminou M.S.M., Liu Y.T., Tan L.Y. (2022). Diabetic retinopathy: An overview of treatments. Indian J. Endocrinol. Metab..

[40] Brownlee M. (2001). Biochemistry and molecular cell biology of diabetic complications. Nature.

[41] Simo R., Hernandez C. (2014). Neurodegeneration in the diabetic eye: New insights and therapeutic perspectives. Trends Endocrinol. Metab..

[42] Pulido J.E., Flamme-Wiese M.J., Leung K., Gao J., Ward L., Wu S.M., Miller D., Schwartz D.M. (2007). A role for excitatory amino acids in diabetic eye disease. Exp. Diabetes Res..

[43] Kusari J., Zhou S.X., Padillo E., Clarke K.G., Gil D.W. (2010). Inhibition of vitreoretinal VEGF elevation and blood–retinal barrier breakdown in streptozotocin-induced diabetic rats by brimonidine. Investig. Ophthalmol. Vis. Sci..

[44] Zhang S.X., Wang J.J., Gao G., Parke K., Ma J.X. (2006). Pigment epithelium-derived factor downregulates vascular endothelial growth factor (VEGF) expression and inhibits VEGF–VEGF receptor 2 binding in diabetic retinopathy. J. Mol. Endocrinol..

[45] Janani R., Anitha R.E., Perumal M.K., Divya P., Baskaran V. (2021). Astaxanthin mediated regulation of VEGF through HIF1α and XBP1 signaling pathway: An insight from ARPE-19 cells and streptozotocin-mediated diabetic rat model. Exp. Eye Res..

[46] Antonetti D.A., Barber A.J., Hollinger L.A., Wolpert E.B., Gardner T.W. (1999). Vascular endothelial growth factor induces rapid phosphorylation of tight junction proteins occludin and zonula occludens-1: A potential mechanism for vascular permeability in diabetic retinopathy and tumors. J. Biol. Chem..

[47] Wang W., Lo A.C.Y. (2018). Diabetic retinopathy: Pathophysiology and treatments. Int. J. Mol. Sci..

[48] Beltramo E., Porta M. (2013). Pericyte loss in diabetic retinopathy: Mechanisms and consequences. Curr. Med. Chem..

[49] Naruse K., Nakamura J., Hamada Y., Nakayama M., Chaya S., Komori T., Kato K., Kasuya Y., Miwa K., Hotta N. (2000). Aldose reductase inhibition prevents glucose-induced apoptosis in cultured bovine retinal microvascular pericytes. Exp. Eye Res..

[50] Ejaz S., Chekarova I., Ejaz A., Sohail A., Lim C.W. (2008). Importance of pericytes and mechanisms of pericyte loss during diabetic retinopathy. Diabetes Obes. Metab..

[51] Frank R.N. (2004). Diabetic retinopathy. N. Engl. J. Med..

[52] Suzuki Y., Nakazawa M., Suzuki K., Yamazaki H., Miyagawa Y. (2011). Expression profiles of cytokines and chemokines in vitreous fluid in diabetic retinopathy and central retinal vein occlusion. Jpn. J. Ophthalmol..

[53] Boss J.D., Singh P.K., Pandya H.K., Tosi J., Kim C., Tewari A., Juzych M.S., Abrams G.W., Kumar A. (2017). Assessment of neurotrophins and inflammatory mediators in vitreous of patients with diabetic retinopathy. Investig. Ophthalmol. Vis. Sci..

[54] Gao F., Hou H., Liang H., Weinreb R.N., Wang H., Wang Y. (2016). Bone marrow-derived cells in ocular neovascularization: Contribution and mechanisms. Angiogenesis.

[55] Chaudhary S., Zaveri J., Becker N. (2021). Proliferative diabetic retinopathy (PDR). Disease-a-Month.

[56] Ciorba A.L., Saber S., Abdelhamid A.M., Keshk N., Elnaghy F., Elmorsy E.A., Abu-Khudir R., Hamad R.S., Abdel-Reheim M.A., Farrag A.A. (2025). Diabetic retinopathy in focus: Update on treatment advances, pharmaceutical approaches, and new technologies. Eur. J. Pharm. Sci..

[57] Wang Z., Zhang N., Lin P., Xing Y., Yang N. (2024). Recent advances in the treatment and delivery system of diabetic retinopathy. Front. Endocrinol..

[58] Lam R.F., Radke N.V., Bandello F.M., Bhende P., Chen Y.T., Cheung G.C.M., Chhablani J., Fung A.T., Hsieh Y.T., Lai C.C. (2025). International consensuses and guidelines on management of proliferative diabetic retinopathy (PDR) by the Asia-Pacific Vitreo-retina Society (APVRS), the Academy of Asia-Pacific Professors of Ophthalmology (AAPPO) and the Academia Retina Internationalis (ARI). Asia Pac. J. Ophthalmol..

[59] Brown D.M., Nguyen Q.D., Marcus D.M., Boyer D.S., Patel S., Feiner L., Schlottmann P.G., Rundle A.C., Zhang J., Rubio R.G. (2013). Long-term outcomes of ranibizumab therapy for diabetic macular edema: The 36-month results from two phase III trials: RISE and RIDE. Ophthalmology.

[60] Nguyen Q.D., Brown D.M., Marcus D.M., Boyer D.S., Patel S., Feiner L., Gibson A., Sy J., Rundle A.C., Hopkins J.J. (2012). Ranibizumab for diabetic macular edema: Results from 2 phase III randomized trials: RISE and RIDE. Ophthalmology.

[61] Korobelnik J.F., Do D.V., Schmidt-Erfurth U., Boyer D.S., Holz F.G., Heier J.S., Midena E., Kaiser P.K., Terasaki H., Teske J.T. (2014). Intravitreal aflibercept for diabetic macular edema. Ophthalmology.

[62] Wykoff C.C., Abreu F., Adamis A.P., Basu K., Eichenbaum D.A., Haskova Z., Lin H., Loewenstein A., Mohan S., Pearce I.A. (2022). Efficacy, durability, and safety of intravitreal faricimab with extended dosing up to every 16 weeks in patients with diabetic macular oedema (YOSEMITE and RHINE): Two randomised, double-masked, phase 3 trials. Lancet.

[63] Zarbin M., Tabano D., Ahmed A., Amador M., Ding A., Holekamp N., Lu X.Y., Stoilov I., Yang M. (2024). Efficacy of Faricimab versus Aflibercept in Diabetic Macular Edema in the 20/50 or Worse Vision Subgroup in Phase III YOSEMITE and RHINE Trials. Ophthalmology.

[64] Gao M., Liu C., Zeng Y., Wu X., Duan J. (2025). Progress in the treatment of diabetic macular edema with faricimab: A review. Front. Med..

[65] Olvera-Barrios A., Lilaonitkul W., Heeren T.F.C., Rozenberg A., Thomas D., Warwick A., Soomro T., Alsaedi A., Schwartz R., Chakravarthy U. (2025). Impact of anti-VEGF treatment for diabetic macular oedema on progression to proliferative diabetic retinopathy: Data-driven insights from a multicentre study. BMJ Open Ophthalmol..

[66] Wong T.Y., Haskova Z., Asik K., Baumal C.R., Csaky K.G., Eter N., Ives J.A., Jaffe G.J., Korobelnik J.F., Lin H. (2024). Faricimab Treat-and-Extend for Diabetic Macular Edema: Two-Year Results from the Randomized Phase 3 YOSEMITE and RHINE Trials. Ophthalmology.

[67] Esteban-Floría O., Mateo J., Lara J., Bartolomé I., Herrero I., Pérez M.A., Cabello C., Honrubia A., Pinilla I., Ascaso J. (2025). Efficacy and safety of faricimab in diabetic macular edema: Real-world outcomes in treatment-naïve and previously treated eyes. J. Clin. Med..

[68] Pieramici D.J., Awh C.C., Chang M., Emanuelli A., Holekamp N.M., Hu A.Y., Suñer I.J., Wykoff C.C., Brittain C., Howard D. (2025). Port Delivery System With Ranibizumab vs Monitoring in Nonproliferative Diabetic Retinopathy Without Macular Edema: The Pavilion Randomized Clinical Trial. JAMA Ophthalmol..

[69] Lowater S.J., Grauslund J., Subhi Y., Vergmann A.S. (2024). Clinical Trials and Future Outlooks of the Port Delivery System with Ranibizumab: A Narrative Review. Ophthalmol. Ther..

[70] Kąpa M., Koryciarz I., Kustosik N., Jurowski P., Pniakowska Z. (2025). Future Directions in Diabetic Retinopathy Treatment: Stem Cell Therapy, Nanotechnology, and PPARα Modulation. J. Clin. Med..

[71] Wang S., Hua R., Zhao Y., Liu L. (2024). Laser Treatment for Diabetic Retinopathy: History, Mechanism, and Novel Technologies. J. Clin. Med..

[72] Chen S.N., Chen S.J., Wu T.T., Wu W.C., Yang C.H., Yang C.M. (2023). Refining vitrectomy for proliferative diabetic retinopathy. Graefes Arch. Clin. Exp. Ophthalmol..

[73] Shaikh N., Kumar V., Ramachandran A., Venkatesh R., Tekchandani U., Tyagi M., Jayadev C., Dogra M., Chawla R. (2024). Vitrectomy for cases of diabetic retinopathy. Indian J. Ophthalmol..

[74] Rong L., Wei W., Fang Y., Liu Y., Gao T., Wang L., Hao J., Gu X., Wu J., Wu W. (2024). Clinical-grade human embryonic stem cell-derived mesenchymal stromal cells ameliorate diabetic retinopathy in db/db mice. Cytotherapy.

[75] Sun F., Sun Y., Wu F., Xu W., Qian H. (2022). Mesenchymal Stem Cell-Derived Extracellular Vesicles: A Potential Therapy for Diabetes Mellitus and Diabetic Complications. Pharmaceutics.

[76] Stitt A.W., Curtis T.M., Chen M., Medina R.J., McKay G.J., Jenkins A., Gardiner T.A., Lyons T.J., Hammes H.P., Simó R. (2016). The progress in understanding and treatment of diabetic retinopathy. Prog. Retin. Eye Res..

[77] Welsh J.A., Goberdhan D.C.I., O’Driscoll L., Buzas E.I., Blenkiron C., Bussolati B., Cai H., Di Vizio D., Driedonks T.A.P., Erdbrügger U. (2024). Minimal information for studies of extracellular vesicles (MISEV2023): From basic to advanced approaches. J. Extracell. Vesicles..

[78] Lintsen D., Broux B. (2026). Effects and mechanisms of adipose tissue-derived extracellular vesicles in vascular inflammation and dysfunction. Neural Regen. Res..

[79] Wu S., Zhang Y., Hou Y., Zhu J., Yang H., Cui Y. (2024). Research on the role of exosomes secreted by immortalized adipose-derived mesenchymal stem cells differentiated into pericytes in the repair of high glucose-induced retinal vascular endothelial cell damage. Exp. Eye Res..

[80] Sharma K., Zhang Y., Paudel K.R., Kachelmeier A., Hansbro P.M., Shi X. (2022). The emerging role of pericyte-derived extracellular vesicles in vascular and neurological health. Cells.

[81] Aheget H., Mazini L., Martin F., Belqat B., Marchal J.A., Benabdellah K. (2020). Exosomes: Their role in pathogenesis, diagnosis and treatment of diseases. Cancers.

[82] Guan J., Meng F., Wang C., Zhang B., Chen J., Han J. (2025). Recent advances in engineered exosome-based therapies for ocular vascular disease. J. Nanobiotechnol..

[83] Fan Y., Chen Z., Zhang M. (2022). Role of exosomes in the pathogenesis, diagnosis, and treatment of central nervous system diseases. J. Transl. Med..

[84] Dairov A., Issabekova A., Ogay V. (2026). Mesenchymal stem cell-derived exosomes in the treatment of skin and subcutaneous tissue diseases: A review. Curr. Stem Cell Res. Ther..

[85] Jiao Y.R., Chen K.X., Tang X., Tang Y.L., Yang H.L., Yin Y.L., Li C.J. (2024). Exosomes derived from mesenchymal stem cells in diabetes and diabetic complications. Cell Death Dis..

[86] Zhang W., Mu Y., Kong Y. (2025). Exosomes derived from mesenchymal stem cells mediate miR-218 and inhibit proliferation, migration, and oxidative stress in retinal vascular endothelial cells via the EGFR/Akt/mTOR signaling pathway. Medicine.

[87] Gao X., He G.H., Zhang X.T., Chen S. (2021). Protective effect of human umbilical cord mesenchymal stem cell-derived exosomes on rat retinal neurons in hyperglycemia through the brain-derived neurotrophic factor/TrkB pathway. Int. J. Ophthalmol..

[88] Cao X., Xue L.-D., Di Y., Li T., Tian Y.-J., Song Y. (2021). MSC-derived exosomal lncRNA SNHG7 suppresses endothelial-mesenchymal transition and tube formation in diabetic retinopathy via miR-34a-5p/XBP1 axis. Life Sci..

[89] Nandeesh V., Zhang X., Zheng K., Asfiya R., Anjugam P., Akpabli-Tsigbe N.D.K., Diaz V., Nguyen T.T., Mooney B., Srivastava A. (2025). Small extracellular vesicles derived from human retinal pericytes under high glucose and hypoxia conditions promote endothelial cell dysfunction in vitro. Exp. Eye Res..

[90] Lupo G., Agafonova A., Cosentino A., Giurdanella G., Mannino G., Lo Furno D., Romano I.R., Giuffrida R., D’Angeli F., Anfuso C.D. (2023). Protective Effects of Human Pericyte-like Adipose-Derived Mesenchymal Stem Cells on Human Retinal Endothelial Cells in an In Vitro Model of Diabetic Retinopathy: Evidence for Autologous Cell Therapy. Int. J. Mol. Sci..

[91] Rajashekhar G., Ramadan A., Abburi C., Callaghan B., O Traktuev D., Evans-Molina C., Maturi R., Harris A., Kern T.S., March K.L. (2014). Regenerative therapeutic potential of adipose stromal cells in early stage diabetic retinopathy. PLoS ONE.

[92] Mendel T.A., Clabough E.B.D., Kao D.S., Demidova-Rice T.N., Durham J.T., Zotter B.C., A Seaman S., Cronk S.M., Rakoczy E.P., Katz A.J. (2013). Pericytes derived from adipose-derived stem cells protect against retinal vasculopathy. PLoS ONE.

[93] Terlizzi V., Kolibabka M., Burgess J.K., Hammes H.P., Harmsen M.C. (2018). The pericytic phenotype of adipose tissue-derived stromal cells is promoted by NOTCH2. Stem Cells.

[94] Yazdanyar A., Zhang P., Dolf C., Smit-McBride Z., Cary W., Nolta J.A., Zawadzki R.J., Marsh-Armstrong N., Park S.S. (2020). Effects of intravitreal injection of human CD34+ bone marrow stem cells in a murine model of diabetic retinopathy. Exp. Eye Res..

[95] Sun F., Sun Y., Zhu J., Wang X., Ji C., Zhang J., Chen S., Yu Y., Xu W., Qian H. (2022). Mesenchymal stem cells-derived small extracellular vesicles alleviate diabetic retinopathy by delivering NEDD4. Stem Cell Res. Ther..

[96] Gu C., Zhang H., Gao Y. (2021). Adipose mesenchymal stem cells-secreted extracellular vesicles containing microRNA-192 delays diabetic retinopathy by targeting ITGA1. J. Cell. Physiol..

[97] Mathieu M., Martin-Jaular L., Lavieu G., Théry C. (2019). Specificities of secretion and uptake of exosomes and other extracellular vesicles for cell-to-cell communication. Nat. Cell Biol..

[98] Pegtel D.M., Gould S.J. (2019). Exosomes. Annu. Rev. Biochem..

[99] Théry C., Witwer K.W., Aikawa E., Alcaraz M.J., Anderson J.D., Andriantsitohaina R., Antoniou A., Arab T., Archer F., Atkin-Smith G.K. (2018). Minimal information for studies of extracellular vesicles 2018 (MISEV2018): A position statement of the International Society for Extracellular Vesicles and update of the MISEV2014 guidelines. J. Extracell. Vesicles.

[100] Coco-Martin R.M., Pastor-Idoate S., Pastor J.C. (2021). Cell Replacement Therapy for Retinal and Optic Nerve Diseases: Cell Sources, Clinical Trials and Challenges. Pharmaceutics.

[101] Salvetat M.L., Pellegrini F., Spadea L., Salati C., Zeppieri M. (2023). Non-Arteritic Anterior Ischemic Optic Neuropathy (NA-AION): A Comprehensive Overview. Vision.

[102] Bianco L., Arrigo A., Aragona E., Antropoli A., Berni A., Saladino A., Parodi M.B., Bandello F. (2022). Neuroinflammation and neurodegeneration in diabetic retinopathy. Front. Aging Neurosci..

[103] Hang A., Feldman S., Amin A.P., Ochoa J.A.R., Park S.S. (2023). Intravitreal Anti-Vascular Endothelial Growth Factor Therapies for Retinal Disorders. Pharmaceuticals.

[104] Gowda A., Wood J.P.M., Chidlow G., Casson R.J. (2026). Laser-induced histopathological changes to the retina: A review. Surv. Ophthalmol..

[105] Rajalakshmi R., Prathiba V., Mohan V. (2016). Does tight control of systemic factors help in the management of diabetic retinopathy?. Indian J. Ophthalmol..

[106] Hoang D.M., Pham P.T., Bach T.Q., Ngo A.T.L., Nguyen Q.T., Phan T.T.K., Nguyen G.H., Le P.T.T., Hoang V.T., Forsyth N.R. (2022). Stem cell-based therapy for human diseases. Signal Transduct. Target. Ther..

[107] Gu X., Yu X., Zhao C., Duan P., Zhao T., Liu Y., Li S., Yang Z., Li Y., Qian C. (2018). Efficacy and Safety of Autologous Bone Marrow Mesenchymal Stem Cell Transplantation in Patients with Diabetic Retinopathy. Cell. Physiol. Biochem..

[108] (2012). A Pilot Clinical Trial of the Feasibility and Safety of Intravitreal Autologous Adult Bone Marrow Stem Cells in Treating Eyes with Vision Loss From Retinopathy. https://clinicaltrials.gov/study/NCT01736059.

[109] (2018). Human iPSC for Repair of Vasodegenerative Vessels in Diabetic Retinopathy. https://clinicaltrials.gov/study/NCT03403699.

[110] (2009). A Comparison of Islet Cell Transplantation with Medical Therapy on the Risk of Progression of Diabetic Retinopathy and Diabetic Macular Edema. https://clinicaltrials.gov/study/NCT00853424.

[111] (2021). Safety of Cultured Allogeneic Adult Umbilical Cord Derived Mesenchymal Stem Cells for the Treatment of Non-Arteritic Ischemic Optic Neuropathy. https://clinicaltrials.gov/study/NCT05147701.

[112] (2023). Proteomic Study of Plasma Exosomes in Patients with Diabetic Retinopathy. https://clinicaltrials.gov/study/NCT06188013.

[113] (2023). Study on Exosome Changes in Patients with Proliferative Diabetic Retinopathy. https://clinicaltrials.gov/study/NCT06198543.

[114] (2017). Prognostic Role of Serum Exosomal miRNA and Its Function in Pathogenesis of Diabetic Retinopathy (DR). https://clinicaltrials.gov/study/NCT03264976.

[115] Mortuza R., Feng B., Chakrabarti S. (2014). miR-195 regulates SIRT1-mediated changes in diabetic retinopathy. Diabetologia.

[116] Yang W., Yan H. (2016). MicroRNA-126 contributes to Niaspan treatment induced vascular restoration after diabetic retinopathy. Sci. Rep..

